# Using an expert survey and user feedback to construct PRECHECK: A checklist to evaluate preprints on COVID-19 and beyond

**DOI:** 10.12688/f1000research.129814.3

**Published:** 2024-06-03

**Authors:** Nora Turoman, Rachel Heyard, Simon Schwab, Eva Furrer, Evie Vergauwe, Leonhard Held

**Affiliations:** 1 Faculty of Psychology and Educational Sciences, University of Geneva, Geneva, Switzerland; 2 Center for Reproducible Science (CRS), University of Zurich, Zurich, Switzerland; 3 Department of Biostatistics at the Epidemiology Biostatistics and Prevention Institute (EPBI), University of Zurich, Zurich, Switzerland; 4 Geneva University Neurocenter, University of Geneva, Geneva, Switzerland

**Keywords:** COVID-19, preprints, checklist, science education, science communication

## Abstract

**Background:**

The quality of COVID-19 preprints should be considered with great care, as their contents can influence public policy. Surprisingly little has been done to calibrate the public’s evaluation of preprints and their contents. The PRECHECK project aimed to generate a tool to teach and guide scientifically literate non-experts to critically evaluate preprints, on COVID-19 and beyond.

**Methods:**

To create a checklist, we applied a four-step procedure consisting of an initial internal review, an external review by a pool of experts (methodologists, meta-researchers/experts on preprints, journal editors, and science journalists), a final internal review, and a Preliminary implementation stage. For the external review step, experts rated the relevance of each element of the checklist on five-point Likert scales, and provided written feedback. After each internal review round, we applied the checklist on a small set of high-quality preprints from an online list of milestone research works on COVID-19 and low-quality preprints, which were eventually retracted, to verify whether the checklist can discriminate between the two categories.

**Results:**

At the external review step, 26 of the 54 contacted experts responded. The final checklist contained four elements (Research question, study type, transparency and integrity, and limitations), with ‘superficial’ and ‘deep’ evaluation levels. When using both levels, the checklist was effective at discriminating a small set of high- and low-quality preprints. Its usability for assessment and discussion of preprints was confirmed in workshops with Bachelors students in Psychology and Medicine, and science journalists.

**Conclusions:**

We created a simple, easy-to-use tool for helping scientifically literate non-experts navigate preprints with a critical mind and facilitate discussions within, for example, a beginner-level lecture on research methods. We believe that our checklist has potential to help guide decisions about the quality of preprints on COVID-19 in our target audience and that this extends beyond COVID-19.

## Introduction

During the COVID-19 pandemic, there has been both a proliferation of scientific data, and a major shift in how results were disseminated, with many researchers opting to post their work as preprints ahead of or instead of publication in scientific journals. ^1^^–^^5^ Preprints are scientific manuscripts that are posted on freely accessible preprint servers (such as medRxiv, bioRxiv, PsyArXiv, MetaArXiv or arXiv) which have not gone through formal peer review. They take between 24 and 48 hours to become ‘live’ – after basic checks by server administrators, ensuring the content of the manuscript is scientific text within the scope of the server, and not spam or plagiarised. ^6^ This is clearly advantageous in a rapidly-evolving pandemic. ^7^^,^^8^ However, unlike journal submissions, the dissemination of preprints is not predicated on any quality control procedure. ^9^ Even before the pandemic, concerns have been raised about the potential of such unvetted results and interpretation of findings leading to widespread misinformation. ^10^ Indeed, we have seen examples of non-peer-reviewed COVID-19-related claims ^11^^,^^12^ that were promptly uncovered as misleading or seriously flawed by the scientific community, ^13^^,^^14^ nonetheless infiltrate the public consciousness ^15^ and even public policy. ^16^ In this high-stakes context, two things have become clear: 1) that preprints have become a tool for disseminating disease outbreak research, ^17^ and 2) that evaluating preprint quality will remain key for ensuring positive public health outcomes.

**Figure 1.**
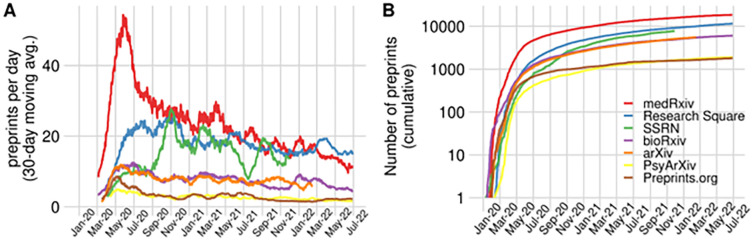
The increase of COVID-19 preprints. (A) Preprints appearing per day on a selection of preprint servers indicated in the figure’s legend, from January 2020 to June 2022. To account for daily variation in the upload of preprints, 30-day moving averages are represented, i.e. the average of the last 30 days. (B) The cumulative number of preprints posted to the same set of preprint servers, indexed in Europe PMC since the first WHO statement regarding a novel infectious disease outbreak on January 9, 2020. ^68^

Since the start of the pandemic, the number of preprints on COVID-19 has been steadily rising, with over 55,000 preprints to date (as of June 27, 2022; see also Figure 1). This proliferation can be explained by researchers hesitating less to upload their results as preprints, following the joint commitment to openly share COVID-19-related content by preprint servers, research funders, universities, scientific societies, private companies, and scientific publishers, the day after the World Health Organization declared the COVID-19 outbreak as a public-health emergency of international concern ( https://wellcome.org/press-release/sharing-research-data-and-findings-relevant-novel-coronavirus-ncov-outbreak; https://wellcome.org/press-release/publishers-make-coronavirus-covid-19-content-freely-available-and-reusable). In the early stages of the pandemic, studies have shown that COVID-19 preprints were typically less well-written in terms of readability and spelling correctness, ^18^ and that most did not meet standards for reproducibility and research integrity. ^19^^–^^21^ A number of preprints also contained extremely serious issues, such as ethical and privacy concerns, data manipulation, and flawed designs. ^22^ These data support the notion of a proliferation of bad quality work over the course of the pandemic (‘research waste’, ^23^^,^^24^), ultimately leading to a spread of misinformation. ^25^

Such initial findings are countered by a much more nuanced story of COVID-19 preprint quality. While it is true that many preprints never convert into publications ^25^^,^^26^ and that preprints tend to be less cited than the resulting peer reviewed publication, ^27^^,^^28^ this is not necessarily due to poor quality. The link between a preprint and its publication may be lost when it is the preprint that gets cited. ^29^ Some authors decide to avoid the publication process altogether, ^30^ or use preprints to release replications, and null results that are difficult to publish, ^31^ or works in progress which may be less well-written and inadequate at sharing data/code. ^18^^–^^21^ Many preprints actually report their results in a balanced way so as not to ‘oversell’ their findings, ^30^^,^^31^ and there is growing evidence of high concordance between findings published in preprints and in peer-reviewed journals. ^25^^,^^32^^–^^39^ Nonetheless, a large portion of COVID-19 preprints show substantial changes in methods and results after peer-review. At least two potential solutions for distinguishing high- from low-quality research in preprints are possible: 1) introducing quality control measures, and 2) educating the readership of preprints to make quality evaluations themselves. Of the extant efforts to improve preprint quality, most have focused on introducing quality control via quick peer-review, e.g., Prereview ( https://www.prereview.org/), Review Commons ( https://www.reviewcommons.org/), PreLights ( https://prelights.biologists.com/), and Rapid Reviews/COVID-19 ( https://rrid.mitpress.mit.edu/rrc19) [ 1]. Though peer-review is often considered the gold-standard for scientific quality control, it has limitations: it can be time consuming, ^3^ at times inefficient at weeding out fraud, and often contaminated with reviewer bias, negligence, and self-interest. ^39^^–^^45^ Automated problem detectors are a promising way forward, ^46^ however such tools still require continued refinement and human verification of results. ^47^ When research is under a stress test, such as during a world-wide pandemic, alternative forms of quality control have to be considered.

Aside from improving the contents of preprints directly, more could be done to educate the readership of preprints on the judicious interpretation of their contents. Preprints receive large attention on social and traditional media, ^48^ but so far, their contents have not always been reported adequately, as many news reports do not provide explanations of the publication process, of how preprint platforms work, and of the implications of non-peer-reviewed research. ^44^^,^^45^^,^^49^^,^^50^ Recent findings show that a simple, one-paragraph explanation of the nature of preprints and the scientific publishing process can change laypeople’s perceived credibility of scientific results, ^51^ while it is unsure whether laypeople actually understand the concepts. ^52^

Public education initiatives on understanding preprints (on COVID-19 or more generally) have been next to non-existent. Apart from the study by Wingen *et al.*, ^51^ only one effort was identified, ^53^ which aimed to provide a set of guidelines for providing feedback on preprints, whether for reviewers or members of the broader community. In the absence of a set of guidelines on interpreting and evaluating information in preprints, we created the PRECHECK project ( www.precheck.site). As the first project of its kind, the aim was to develop a simple, user-friendly tool to guide non-scientists in their evaluation of preprint quality. Though we were inspired by the proliferation of preprints and misinformation during the COVID-19 pandemic, we created a tool that can be applied to preprints or publications on other empirical research topics including human subjects, with hopes that it can help empower non-scientists and non-specialists in making their own judgments.

## Methods

### Ethical considerations

The entire project “PRECHECK: A checklist to evaluate COVID-19 preprints” was conducted under the ethical policies of the University of Zurich. As stipulated in sections 5 and 6 of these policies ( https://www.rud.uzh.ch/dam/jcr:c42f07d3-2e89-485c-8a8b-3a3c3f46a3f5/UZH%20Policy%20on%20the%20Ethical%20Review%20of%20Research%20Projects%20Involving%20Human%20Subjects%20 (UZH%20Ethics%20Policy).pdf), our project falls outside the scope of the Swiss Human Research Act and, per section 8.1 of these policies, can be considered as a study that “generally cannot harmfully affect study participants”. Therefore, our project did not require explicit prior ethical approval from the institution, and for this reason we did not ask for ethical approval from the institution. A post-hoc verification with the University of Zurich ethical commission’s ethics review checklist (available in in the OSF repository for this project ^54^) confirmed that we did not require ethical approval from the current project per the regulations of the University of Zurich. Written consent for participating in student workshops was not required because the workshops were administered as part of their regular university courses (details in manuscript) to which students do not need to exceptionally consent. Moreover, no data was collected from students, nor was it incorporated in the PRECHECK checklist. There was no relationship of dependence between the students and the workshop presenters (i.e., they were not involved in the grading of the students’ course assignments or exams). In the workshops for journalists, some participants offered unsolicited feedback; these participants were aware that their feedback would be used for this purpose through the presentation given during the workshop.

### Study design

**Figure 2.**
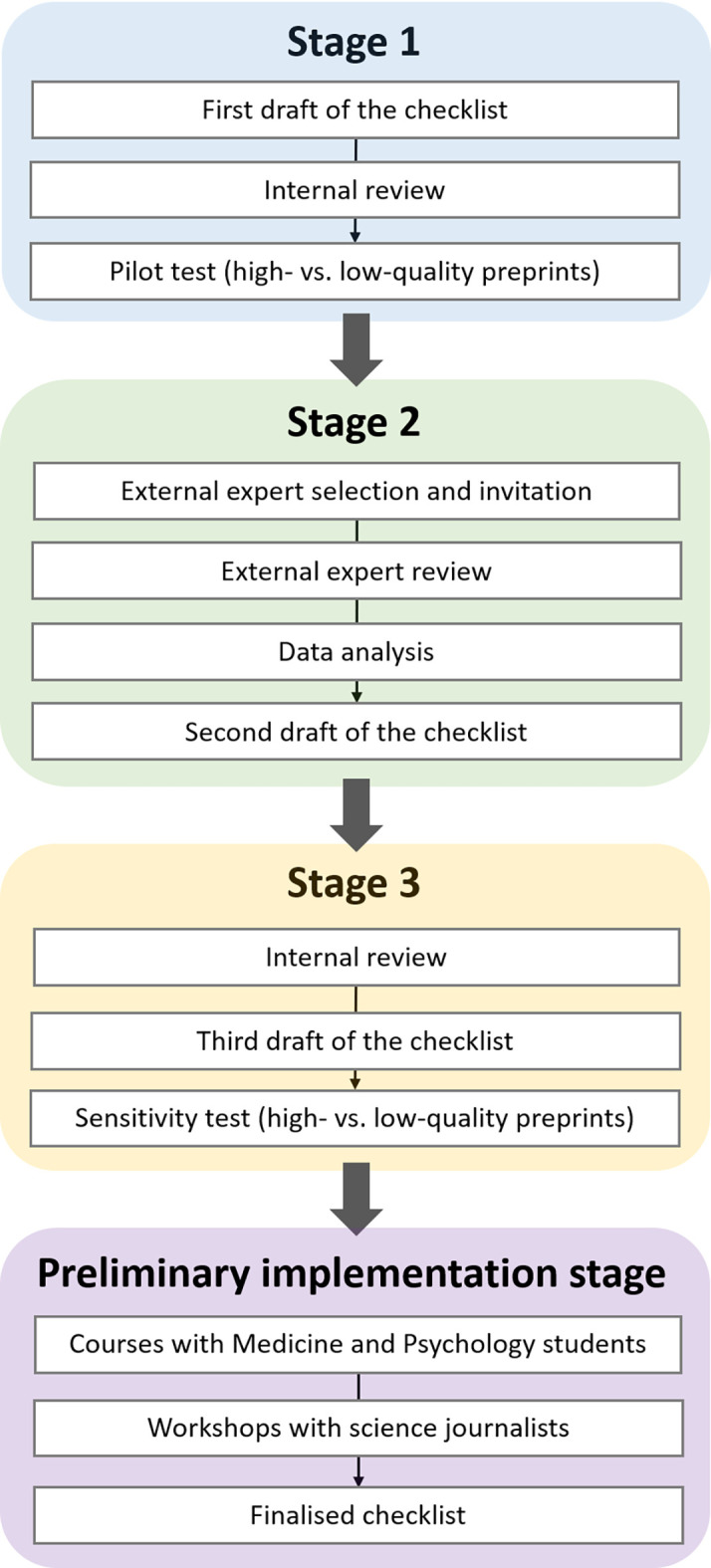
The stages of our Delphi-inspired approach to creating the PRECHECK checklist. There are four successive stages (Stage 1 – Stage 3 and the implementation stage, in blue, green, yellow, and purple fields, respectively) where each is made up of a set of successive steps (in white rectangular fields).

The aim of the study was to develop simple and clear guidance, in the form of a checklist, to help assess the quality of a preprint. Our target audience for the checklist were scientifically literate non-specialists, such as students of medicine and psychology, and science journalists. These two target groups were selected, first, for convenience since we had direct access to them. Second, as detailed in the introduction, the COVID-19 pandemic and its flood of research was the primary motivation for the development of the checklist and science journalists were the logical target group to address. Third, our student populations had also been exposed to preprints more intensively during the COVID-19 pandemic and we targeted them in our educational mandate. To develop a checklist that would be both user-appropriate, and discriminative of preprints of different levels of quality, we applied a multi-step approach inspired by the qualitative Delphi method. ^55^^,^^56^ As such, our study could be considered a qualitative study, and we have thus followed the Standards for Reporting Qualitative Research (SRQR) (Ref. 57; see Ref. 54 for a completed version of the checklist for the current project). The Delphi method uses successive voting by expert groups to iteratively narrow down a pool of options until consensus is reached and is effective in determining consensus in situations with little to no objective evidence. ^58^ Our procedure involved four main stages. The first draft of the checklist was developed by the junior members of the team after an initial review of the literature and potential solutions (which forms the introduction of this paper). In the first stage, this draft was reviewed internally by the senior members of our team (who were not involved in creating the draft) and subjected to pilot testing to verify whether the checklist could discriminate high- from low-quality preprints [ 2] if used by experts. In the second stage, a panel of external experts rated the relevance of each element of the checklist and provided feedback, after which the checklist was updated. In the third stage, we conducted a final round of internal review producing the third draft of the checklist, which was also submitted to a final pilot testing. At the end of the above three stages, we verified if members of our target audience could use the checklist successfully and if they appeared to find it useful, via workshops with university students and journalists. We called this stage the Preliminary implementation of the checklist. In the workshop for journalists, some participants offered unsolicited yet helpful feedback, which was incorporated into the finalised version of the checklist (see also Figure 2). These participants were aware that their feedback would be used for this purpose through the presentation given during the workshop. We caution that the chosen setup of the Preliminary implementation stage does not allow a systematic validation of the checklist, especially since the target group was chosen mainly for convenience.

### Researcher characteristics

The research team conducting the analysis was composed of three junior researchers (postdoctoral level) and three senior researchers (two professors and one senior scientific collaborator). All members of the team have experience in meta-research, but are trained in other disciplines (statistics and psychology).

### Internal review

In this step, the three senior members of our team gave written feedback on a draft of the checklist in the form of comments. After the written feedback was incorporated by two junior members of our team, final feedback in the form of verbal comments from the senior members was obtained in an online meeting round. A copy of each version of the checklist after feedback and other validation procedures below is available in the OSF repository for this project. ^54^

### Pilot testing

After each of the two rounds of internal review (Stage 1 and Stage 3), we conducted pilot testing in order to verify whether the checklist, at its given stage of development, could successfully discriminate between preprints of different levels of quality. We did not perform any statistical analyses as part of this assessment. Since objective criteria for quality that would apply to all preprints across disciplines were difficult to envision, we used a list of milestone research works in the COVID-19 pandemic ^59^ as a proxy of high quality, and we identified what would be the preprints in our high-quality set from this list. The preprints that would make up our low-quality set, on the other hand, were chosen from a list of retracted COVID-19 preprints, ^60^ with retraction being a proxy for low quality. There were no *a-priori* criteria for selecting preprints. We selected three high-quality preprints ^61^^–^^63^ and three low-quality preprints ^11^^,^^64^^,^^65^ to test across multiple stages. Since preprint selection occurred in August 2021, we only included preprints that were available online from the start of the pandemic up until that point. In the Stage 1 round, one junior team member tested only the high-quality preprints and another junior team member the low-quality preprints. In the Stage 3 round, both team members tested all preprints independently. The same high- and low-quality preprints were used at both stages. To document the test results, a spreadsheet was generated with one field per checklist element, where the response to the checklist element for each preprint was entered. Fields were also coloured red, green, and yellow, to visually better represent ‘yes’, ‘no’, and ‘maybe’ responses to specific checklist elements, respectively. The results of each round of pilot testing with the links to the respective preprints are available in the Open Science Framework (OSF) repository for this project. ^54^ Due to the limited number of assessed preprints, only a qualitative summary of the results is possible and no statistical analysis of these results is provided.

### External expert review

*Panel selection*

We invited a total of 54 experts across four topic groups: research methodology (14 invitations), preprints (15 invitations), journal publishing (15 invitations), and science journalists (10 invitations), as these fields of expertise were judged to be of the greatest relevance for evaluating our checklist. Experts were identified through personal connections, by identifying editors of relevant journals in the fields of psychology and medicine (for the editor cohort), science journalists from a list of speakers at the World Conference of Science Journalists, Lausanne 2019 ( https://www.wcsj2019.eu/speakers); for the science journalism group), and identifying individuals whose work in the topic areas of research methodology and preprints is noteworthy and well-known. Thus, we invited science journalists, journal editors, as well as researchers whose research focuses on research methodology and preprints. There were no *a-priori* criteria for selecting experts, other than their perceived belonging to one of the topic groups. In actual Delphi designs, 5-10 experts may be considered sufficient, ^66^ however, there is no clear consensus on how large expert groups should be. ^67^ Of the total number of experts, 29 were personal contacts. Panel members were contacted by email between October 18 2021 and November 16 2021 with a standardised explanation of the project, their role in the refinement of the checklist, how their data would be used (that their name and email will not be shared, that their responses will be fully anonymous, and that the aggregated results will be published and shared on our OSF repository for the project), and a link to a Google Forms survey where they could enter their responses anonymously. By clicking on the link to the survey, the experts thus gave their implicit informed consent to participate. The Survey Form sent to the experts is available in the OSF repository for this project. ^54^ Experts that replied to our email declining to take part in the survey were not contacted further. Any experts that did not explicitly decline to take part were reminded twice, as the survey responses were fully anonymous, and we had no way of linking responses to individual experts. A total of 26 experts (48%) responded to the invitation by filling our survey (14 of them were personal contacts). The experts (self-)reported that they belonged to the following expert groups: four experts in science journalism, four journal editors, seven meta-researchers/experts on preprints, and 11 methodologists.

*Response collection and analysis*

Experts rated the relevance of each element of the checklist on a five-point Likert scale, with the following response options: extremely irrelevant, mostly irrelevant, neither relevant nor irrelevant, mostly relevant, and extremely relevant. Response data were analysed by computing mean responses per element, using R (Version 4.04). We based our decisions on which elements to keep following the procedure used to establish the CONSORT guidelines for abstracts. ^68^ That is, all elements with a mean score of four and above were kept (in the CONSORT for abstracts, this score was eight, as they used a 10-point Likert scale), elements with a mean score between three and four (four not included; between six and seven in the CONSORT guidelines for abstracts criteria) were marked for possible inclusion, and elements with a mean score below three were rejected.

Experts also had the option to provide free-text comments: general additional comments on the checklist elements, suggestions for potentially relevant items that are missing, and suggestions on the structure (a PDF of the survey, including the Likert scales and free-text options is available in the OSF repository for this project ^54^). These comments were collected into a single document and responded to in a point-by-point manner akin to a response to reviewers document (also in the OSF repository for this project ^54^), arguing our agreement/disagreement with expert comments and how they were addressed in the subsequent draft of the checklist.

*Preliminary implementation*

Experts rated the relevance In the Preliminary implementation stage we envisaged two workshops for students: one for Bachelors’ students of Psychology at the University of Geneva, and one for Bachelors’ students of Medicine at the University of Zurich. Both workshops were given as invited lectures in standard classes taught to students as part of their respective Bachelors’ courses (as part of the “Scientific skills and competencies in psychology” class at the University of Geneva [in the original French: “Compétences et connaissances scientifiques en psychologie”], and the “Biostatistics for medical professionals” class at the University of Zurich [in the original German: “Biostatistik für Mediziner”]). The workshop content was made clear to the students in advance. Only those students that were enrolled in the above classes as part of their Bachelors’ courses could attend the workshops. Thus, no special consent was required to participate in the workshops. One junior member led the workshop at the University of Geneva, while at the University of Zurich, one junior and one senior member led the workshop. There was no relationship of dependence between either one of these members and the students they administered the workshops to (i.e., neither member was involved in the grading of the students’ course assignments or exams). Both workshops started with a theoretical presentation of the scientific publishing process, what preprints are, and current issues with preprints and peer review (based on the conclusions of the literature review in the present manuscript). Next, the purpose and component parts of the checklist were presented, together with instructions on how to use it for the subsequent in-class exercise. In the exercise, students were split into two groups according to their last names, each given 15-20 minutes to re-read a preprint (they were informed about the exercise and given the preprints to read before the workshop as homework; one group read, ^64^ and the other group read) ^62^ and use the checklist to evaluate it. The answers were collected anonymously, in an aggregated (non-individual) fashion, via an in-house platform. At the University of Geneva, we used surveys on Votamatic ( https://votamatic.unige.ch/) to gather the percentages of students that voted ‘yes’, ‘no’, or ‘partly’ for each superficial-level item, and Padlet ( https://unige.padlet.org) to gather optional free text responses to the deep-level items. At the University of Zurich, Klicker ( https://www.klicker.uzh.ch) was used to gather the percentages of students that voted ‘yes’, ‘no’, or ‘maybe’ for each superficial-level item, alongside optional free text comments. Once recorded, these percentages were discussed by the whole class, and compared against the researchers’ own evaluation of said preprint. The responses were only analysed inasmuch as percentages of ‘yes’, ‘no’, and ‘partly/maybe’ responses were automatically generated by the respective platforms, during the class, for the purpose of in-class discussion. These responses were not recorded outside of the platform (e.g., on any project team member’s computer), nor were they subject to any formal statistical analyses performed on these responses, and the responses did not influence the content of the checklist in any way. We did not ask any question about the workshop participants’ educational background or level of experience with academic research.

The above workshop format was also adapted for science journalists employed at the Universities of Geneva and Zurich (i.e. the members of the respective Communication Departments). Eventually only the workshop at the University of Geneva was held (and led by the junior member of the team based at the University of Geneva), as the relevant personnel at the University of Zurich did not respond to our requests to hold a workshop. The format was largely the same as that of the workshop for students, except that the initial presentation focused less on the scientific publishing process, and more on issues with preprints and peer review, and included more time for a mutual discussion of said issues. We intended for these to be small workshops open only to a select number of people, on a purely voluntary basis, invited by the main contact person at each University’s communications section. All of the invited parties were informed on the content of the workshop beforehand. Only those individuals that were interested in attending did so and provided verbal assent to participate in the workshop. The same in-house online platform as before (i.e., Votamatic) was used to collect data on percentages of ‘yes’, ‘no’, or ‘partly’ for each superficial-level item of the checklist in an anonymous, aggregated (non-individual) fashion, and this automatic generation of response percentages was the only analysis performed on these data. Deep-level item answers were informally discussed. As before, there were no formal statistical analyses performed on these data. Some informal verbal feedback (not previously solicited) that we received from the participants did influence the content of the checklist, as we detail in the Preliminary implementation section below.

Finally, the content of the workshop for university science journalists was adapted for a fact-checking seminar organised by the Swiss Association of Science Journalists ( https://www.science-journalism.ch/event/spring-seminar-2022). Two junior members gave this workshop at the Swiss National Science Foundation headquarters in Bern. Participation was voluntary and open only to individuals that registered to attend the conference at which we were invited to give our workshop as part of the advertised programme. Thus, no special consent was required for participation. We used the same procedure as above, with the exception that a ‘nonsense’ preprint was read and evaluated; ^69^ i.e., a manuscript describing a made-up study to prove the point that predatory journals do not screen for quality) and no response data were collected (responses were probed and discussed verbally during the session).

## Results

### Stage 1 results

*First draft of the checklist*

The first draft of the checklist was created from April 16 until May 17, 2021, and contained six categories of items: research question, study type, transparency, limitations, study reporting, and research integrity. The research question item asked whether the study mentioned the research question/aim, this being the most basic component of a research study. The study type question asked whether the study type was mentioned, and in the case that it was not, asked users to try and infer what the study type was, with guidance. In transparency, users were supposed to check the existence, availability, and accessibility of a protocol, and of data, code, and materials sharing. The limitations question asked whether any limitations of the study were mentioned, and asked users to try and evaluate any potentially unmentioned limitations (biases, specifically), with guidance. In study reporting, we asked to check for reporting guidelines that were followed explicitly or implicitly. Finally, the research integrity category asked users to check whether ethical approval, conflicts of interest, and contributor roles were reported.

*Internal review results*

In the first round of internal review, the senior members of our team provided feedback on the contents of the first draft of the checklist. This round revealed that explanations were needed as to the importance of considering each specific item in our checklist, and that both a more ‘superficial level’ and a ‘deeper level’ were necessary to account for all user needs. For these reasons, we expanded the initial checklist, such that each item consisted of a main question, an explanatory section entitled ‘Why is this important’, and a section entitled ‘Let’s dig deeper’. The main questions formed the basis of the checklist, as they were all closed questions that could be answered via the ‘yes’ box next to them. Users could tick the box in full to indicate that the preprint being read passes the question, in part to indicate a ‘maybe’ response, or not at all to indicate that the preprint does not pass the question. This level was also called the ‘superficial level’ of assessment, as the questions could mostly be answered after a quick read of a preprint, and by searching for keywords that appear in the questions. The ‘why is this important?’ section was added in order to increase the pedagogical value of the checklist, which was designed as a teaching tool, such that users could learn about the purpose behind the main questions, and their importance for evaluating research. The ‘let’s dig deeper’ sections were added as an optional part for users that wanted to go beyond the main questions in their evaluation of a preprint, or that wanted to learn more about how to structure their thoughts when evaluating research work. Thus, this section does not have a tick-box, as it usually contains open questions and suggestions. This section cannot stand alone and should be consulted after the main questions and ‘why is this important’ section, which is why we called this the ‘deep level’ of assessment. A full version of the checklist at this stage can be found in the OSF repository for this project. ^54^ This was the version of the checklist that we submitted to a pilot testing on high- and low-quality preprints.

*Results of the pilot testing*

While searching for high- and low-quality preprint examples to apply the checklist to, we discovered that the checklist works best when applied to research with human subjects using primary data (or systematic reviews, meta analyses and re-analyses of primary data). That is, the questions turned out to be ill-posed (unanswerable) for research that did not fall into the above description, such as simulation studies, for example. We decided to indicate this in the introduction section of the checklist.

At the superficial level, the high-quality preprints that we used ^61^^–^^63^ received mostly ‘yes’ and ‘maybe’ responses to the main questions. Only ^62^ had a ‘no’ for mentioning/sharing their data/code. The low-quality preprints ^11^^,^^64^^,^^65^ also received several ‘yes’ and ‘maybe’ responses, though fewer than the high-quality preprints. Interestingly, the Transparency and Study reporting items all received ‘no’ responses for the low-quality preprints, whereas the high-quality preprints mostly received ‘maybe’ and ‘yes’ responses. This demonstrates both that high- and low-quality preprints differ in terms of how they meet transparency and reporting standards, but also highlights that even high-quality preprints do not necessarily meet all these standards.

At the deep level, high-quality preprints mostly received ‘yes’ responses to the points in the ‘let’s dig deeper’ section, and a few ‘no’ and ‘maybe’ responses. Meanwhile, the low-quality preprints had no clear pattern of responses with many ‘no’, ‘yes’, and ‘maybe’ responses. However, low-quality preprints had more ‘no’ responses than high-quality preprints. At this level of assessment, one could delve into issues with study design, specifically in Limitations, where low-quality preprints performed demonstrably worse than high-quality preprints. Thus, it may be important to consider both levels of assessment when evaluating preprints using the checklist. The final version of the checklist at this stage was completed on October 18, 2021.

### Stage 2 results

*External expert review summary results*

Experts were first asked how relevant they thought each of the six item categories were for assessing preprint quality on a five-point Likert scale: from extremely irrelevant (score of 1) to extremely relevant (score of 5). Here, all categories scored above four, except study reporting which received a 3.96. Next, experts were to rate the relevance of each of the elements per category, including the main questions, and each of the points in the ‘let’s dig deeper’ sections. The results are summarised in Table 1 below. None of the elements has a score lower than three, which meant that none of the elements warranted immediate exclusion. However, several elements, including the entire study reporting category, had scores between three and four that placed them in the ‘possible inclusion’ category.

**Table 1.** Results of expert survey and implementation of results.

Checklist Element	Average Expert Score (SD)	Decision
1. Research Question	4.23 (1.3)	Keep
1a) Is the research question/aim stated?	4.04 (1.4)	Keep
LDD 1) Is the study confirmatory or exploratory?	3.69 (1.2)	Reject after discussion
2. Study Type	4.31 (0.9)	Keep
2a) Is the study type mentioned in the title, abstract, introduction, or methods?	4.27 (0.8)	Keep
LDD 2) What is the study type?	4.08 (1.1)	Keep
3. Transparency	4.19 (1.0)	Keep
3a) Is a protocol, study plan, or registration mentioned, and accessible (e.g., by hyperlink, by registration number)?	4.04 (1.0)	Keep
3b) Is data sharing mentioned, and are the data accessible (e.g., by hyperlink)?	3.88 (0.9)	Keep after discussion
3c) Is code/materials sharing mentioned, and are they accessible (e.g., by hyperlink)?	3.77 (0.9)	Keep after discussion
LDD 3a) Try to find and read the study protocol - are the procedures described in the protocol consistent with what was reported in the preprint?	4.00 (1.0)	Keep
LDD 3b) Do the same with the shared data - do they match the data reported in the manuscript?	4.15 (0.9)	Keep
4. Limitations	4.19 (1.1)	Keep
4a) Are the limitations of the study addressed in the discussion/conclusion section?	4.19 (1.1)	Keep
LDD 4a) Check the study’s sample (methods section)?	4.50 (1.0)	Keep
LDD 4b) Was there a control group or control condition?	4.19 (1.0)	Keep
LDD 4c) Was there randomisation?	4.08 (1.2)	Keep
LDD 4d) Was there blinding?	4.00 (1.1)	Keep
5. Study Reporting	3.96 (1.1)	Reject after discussion
5a) Were any reporting guidelines used (e.g., PRISMA, SPIRIT, CONSORT, MOOSE, STROBE, STARD, ARRIVE; JARS, in medical and psychological	3.85 (1.1)	Reject after discussion
LDD 5a) Have a look at the criteria of the reporting guidelines that fit the discipline of the manuscript, and try to see if the study follows them even if they do not state it	3.65 (0.9)	Reject after discussion
LDD 5b) Were all the performed analyses reported in the introduction and/or methods section?	3.81 (1.0)	Reject after discussion
LDD 5c) Were all the research questions/aims stated in the introduction addressed by analyses (see results and discussion sections)?	3.88 (1.1)	Reject after discussion
LDD 5d) Were exploratory analyses stated as exploratory (e.g., in the introduction section)?	3.69 (1.1)	Reject after discussion
6. Research Integrity	4.08 (1.1)	Keep
6a) Does the article contain an ethics approval statement (e.g., approval granted or no approval required), that can be verified (e.g., by approval number)?	4.04 (1.2)	Keep
6b) Have conflicts of interest been declared?	4.08 (1.3)	Keep

The experts evaluated the relevance of the checklist elements on a five-point Likert scale. The average expert scores are shown here. The higher the score, the more relevant the element. The decisions taken for each element are summarised in the last column. If the element achieved an average expert score higher or equal to four, the element was kept in the checklist and into the next phase. Elements with an average lower than three were rejected; no element was scored that low in relevance. The remaining elements with an average between three and four were discussed to make a final decision. LDD stands for ‘Let’s dig deeper’ and SD for standard deviation.

*Summary of external experts’ comments*

The full list of free-text comments submitted by the experts and our responses to them is available in the OSF repository for this project. ^54^ Overall, the comments revealed enthusiasm about the checklist, but also important criticisms. First, there were concerns that the target audience as it was previously defined (non-scientists) would not be able to adequately use a checklist of the complexity present at the time. Paradoxically, however, there were also many suggestions for additional elements that could further increase complexity, such as: how the study fills the gap in the knowledge, if the study can be fully reproduced, if there is justification for the statistical methodology used, etc. Another prominent criticism was that the checklist inadvertently prioritised randomised controlled trials and could potentially discredit observational studies. Our pilot testing did not find observational studies to be particularly disadvantaged in comparison with randomised controlled trials. However, we did acknowledge that some categories, specifically limitations, appeared more oriented towards randomised controlled trials. One specific comment raised the issue that our checklist did not include COVID-19 specific questions, despite the checklist’s motivation. We thus sought to correct and elaborate these points in the next versions of the checklist.

Some experts had additional ideas, some of which were outside the scope of this project, but others of which were easy to implement. In particular, the suggestion to ask users to check if the manuscript converted into a publication, and to include spin and overinterpretation of results as other examples of biases in the Limitations section. These suggestions were incorporated into the subsequent versions of the checklist.

*Second draft of the checklist*

The second draft of the checklist ^54^ consisted of the following elements: Research question (main question only), study type, transparency (main question on mentioning the protocol, and two ‘let’s dig deeper’ questions on accessing the protocol, and accessing the data), limitations, and research integrity (two main questions on mentioning an ethics approval statement, and conflicts of interest). After expert feedback, we decided to omit the study reporting category, and instead incorporate the reporting guidelines mentioned there in the newly added descriptions of study types in the item study type, to reduce overall complexity, and increase clarity in this section specifically. After comments on the checklist’s complexity, we combined the transparency and research integrity categories and their remaining elements into a new category called ‘transparency and integrity’. Here, in response to a specific comment, we altered the main questions such that they probe if a study protocol, data sharing, materials sharing, ethical approval, and conflicts of interest, are mentioned, whereas the ‘let’s dig deeper’ section asks if the above are accessible (in the aforementioned section, we define what we mean by accessible). In addition to these changes, we realised the utility of looking both at the preprint and the preprint server when using the checklist, as sometimes the preprint does not mention data/materials sharing, but these resources are shared on the server. ^20^ Thus, we added a recommendation into the introduction to consider both sources when using the checklist. In response to the comment on inadvertently disadvantaging observational research, we added disclaimers into the limitations section stating when certain sources of bias do not apply. Specifically, for the control group/condition, randomisation, and blinding elements, we added a clause to the end of each paragraph that states ‘(if your preprint is on an observational study, this item does not apply)’. In response to the lack of a COVID-19 element, we elaborated that, although the state of COVID-19 preprints and their effect on society was our inspiration for the project, our expertise (preprint quality in medicine and psychology) made it difficult to create an adequate COVID-19-specific checklist. With this, we modified the title of the checklist and expanded the introduction, to avoid further confusion. The final version of the checklist at this stage was completed on January 17, 2022. It is available in the OSF repository for this project. ^54^ Also, we structured our workshops with students and journalists around issues with preprints and peer review more generally.

### Stage 3 results

*Final draft of the checklist*

In the second internal review round, we kept the four-item structure, most of the text, and visual separation between the superficial and deep levels from the second draft of the checklist. In addition, the wording was clarified and additional explanations were provided where necessary. In transparency and integrity, we added clauses to explain what to do in situations where data and materials are stated to be shared upon request, or if reasons against sharing are mentioned. Specifically, since acquiring data/materials after requesting it from authors that only share upon request happens only in a minority of cases, ^70^ we advised that ‘mentioning only that data will be shared ‘upon request’ does not count’. However, if authors mention that they cannot share the data or materials for a specific reason, we advised that ‘mentioning any reasons against sharing also counts [as a ‘yes’]’. In the limitation section, we added examples of spin and overinterpretation as other sorts of biases to look out for. In the introduction, we added the recommendation to check whether the preprint has converted into a publication.

*Results of pilot testing*

At the superficial level, both high- and low-quality preprints had many positive responses, for both team members that tested the checklist. The elements that differed the most were transparency and integrity, with low-quality preprints having slightly more ‘no’ and ‘maybe’ responses than high-quality preprints. Additionally, both raters agreed that two of the three low-quality preprints did not discuss limitations. This is a slight departure from the prior pilot testing results, in that the difference between high- and low-quality preprints appears to be smaller.

At the deep level, however, the differences were once again well-pronounced, as high-quality preprints had predominantly positive responses, while low-quality preprints had predominantly negative and ‘maybe’ responses. Thus, the deep level seems to be necessary to fully discern between high- and low-quality preprints. Similarly to the prior results, the responses in the transparency and integrity section exposed that even high-quality preprints did not always meet the standards of transparency and integrity. The final version of the checklist at this stage was completed on March 1, 2022.

### Preliminary implementation

With an operational checklist, we set out to teach members of our target audience how to use the checklist and to verify if they could use it as intended. To this end, we gave courses including workshops to Bachelor students in Medicine and Psychology at the University of Zurich, and the University of Geneva, respectively. The workshop at the University of Zurich took place on March 11, 2022, while the workshop at the University of Geneva took place on May 9, 2022. For our journalist cohort, we provided a lecture and practical for members of the University of Geneva communications office and scientific information division. This workshop took place on May 19, 2022. We later also presented the checklist at a fact-checking seminar organised by the Swiss Association of Science Journalists on May 31, 2022.

Across all of these classes, except for the fact-checking seminar, we used the same high- and low-quality preprints as practice material, and explained both how to use the checklist, as well as the rationale behind its elements, as stated in our point-by-point response to experts. A few suggestions for improvement nonetheless emerged from the workshop with the journalists at the University of Geneva, as some participants wished to offer feedback. Some participants of this workshop pointed out that the necessity and function of both the superficial and deep level of assessment were not clear. Others had trouble understanding what mention of data and materials sharing counts as a ‘yes’ response and what counts as a ‘no’ response. Finally, one participant suggested that even before using the checklist, one should generally apply more caution when assessing controversial research or findings that sound ‘too good to be true’. We incorporated all of this feedback into the final version of the checklist, which was completed on May 20, 2022. ^54^

During post-publication peer-review, it became apparent that our checklist did not address qualitative research at all. Since we have only designed the checklist with quantitative research in mind, we expanded the disclaimer in the Introduction to the checklist to state that it is not optimised for qualitative aspects of research studies. This was the only change to any part of the checklist as a result of peer review, and it resulted in a new version of the checklist completed on December 18, 2023 (see also Table 2). ^54^

**Table 2.** The PRECHECK checklist.

	Category	Items	Yes
Research question	1	Is the research question/aim stated?	□
Why is this important?		A study cannot be done without a research question/aim. A clear and precise research question/aim is necessary for all later decisions on the design of the study. The research question/aim should ideally be part of the abstract and explained in more detail at the end of the introduction.	
Study type	2	Is the study type mentioned in the title, abstract, introduction, or methods?	□
Why is this important?		For a study to be done well and to provide credible results, it has to be planned properly from the start, which includes deciding on the type of study that is best suited to address the research question/aim. There are various types of study (e.g., observational studies, randomised experiments, case studies, etc.), and knowing what type a study was can help to evaluate whether the study was good or not. What is the study type? Some common examples include: - observational studies - studies where the experimental conditions are not manipulated by the researcher and the data are collected as they become available. For example, surveying a large group of people about their symptoms is observational. So is collecting nasal swabs from all patients in a ward, without having allocated them to different pre-designed treatment groups. Analysing data from registries or records is also observational. For more information on what to look for in a preprint on a study of this type, please consult the relevant reporting guidelines: STROBE. - randomised experiments - studies where participants are randomly allocated to different pre-designed experimental conditions (these include Randomised controlled trials [RCTs]). For example, to test the effectiveness of a drug, patients in a ward can be randomly allocated to a group that receives the drug in question, and a group that receives standard treatment, and then followed up for signs of improvement. For more information on what to look for in a preprint on a study of this type, please consult the relevant reporting guidelines: CONSORT. - case studies - studies that report data from a single patient or a single group of patients. For more information on what to look for in a preprint on a study of this type, please consult the relevant reporting guidelines: CARE. - systematic reviews and meta-analyses - summaries of the findings of already existing, independent studies. For more information on what to look for in a preprint on a study of this type, please consult the relevant reporting guidelines: PRISMA.	
Let’s dig deeper		If the study type is not explicitly stated, check whether you can identify the study type after reading the paper. Use the question below for guidance: - Does the study pool the results from multiple previous studies? - If yes, it falls in the category systematic review/meta-analysis. - Does the study compare two or more experimenter-generated conditions or interventions in a randomised manner? - If yes, it is a randomised experiment. - Does the study explore the relationship between characteristics that were not experimenter-generated? - If yes, then it is an observational study - Does the study document one or multiple clinical cases? - If yes, it is a case study.	
Transparency and Integrity	3	(a) Is a protocol, study plan, or registration of the study at hand mentioned? (b) Is data sharing mentioned? Mentioning any reasons against sharing also counts as a ‘yes’. Mentioning only that data will be shared “upon request” counts as a ‘no’. (c) Is materials sharing mentioned? Mentioning any reasons against sharing also counts as a ‘yes’. Mentioning only that materials will be shared “upon request” counts as a ‘no’. (d) Does the article contain an ethics approval statement (e.g., approval granted by institution, or no approval required)? (e) Have conflicts of interest been declared? Declaring that there were none also counts as a ‘yes’.	□ □ □ □ □
Why is this important?		Study protocols, plans, and registrations serve to define a study’s research question, sample, and data collection method. They are usually written before the study is conducted, thus preventing researchers from changing their hypotheses based on their results, which adds credibility. Some study types, like RCT’s, must be registered. Sharing data and materials is good scientific practice which allows people to review what was done in the study, and to try to reproduce the results. Materials refer to the tools used to conduct the study, such as code, chemicals, tests, surveys, statistical software, etc. Sometimes, authors may state that data will be “available upon request”, or during review, but that does not guarantee that they will actually share the data when asked, or after the preprint is published. Before studies are conducted, they must get approval from an ethical review board, which ensures that no harm will come to the study participants and that their rights will not be infringed. Studies that use previously collected data do not normally need ethical approval. Ethical approval statements are normally found in the methods section. Researchers have to declare any conflicts of interest that may have biased the way they conducted their study. For example, the research was perhaps funded by a company that produces the treatment of interest, or the researcher has received payments from that company for consultancy work. If a conflict of interest has not been declared, or if a lack of conflict of interest was declared, but a researcher’s affiliation matches with an intervention used in the study (e.g., the company that produces the drug that is found to be the most effective), that could indicate a potential conflict of interest, and a possible bias in the results. A careful check of the affiliation of the researchers can help identify potential conflicts of interest or other inconsistencies. Conflicts of interests should be declared in a dedicated section along with the contributions of each author to the paper.	
Let’s dig deeper		(a) Can you access the protocol/study plan (e.g., via number or hyperlink) (b) Can you access at least part of the data (e.g., via hyperlink, or on the preprint server). Not applicable in case of a valid reason for not sharing. (c) Can you access at least part of the materials (e.g., via hyperlink, or on the preprint server). Not applicable in case of a valid reason for not sharing. (d) Can the ethical approval be verified (e.g., by number). Not applicable if it is clear that no approval was needed. By ‘access’, we mean whether you can look up and see the actual protocol, data, materials, and ethical approval. If you can, you can also look into whether it matches what is reported in the preprint.	
Limitations	4	Are the limitations of the study addressed in the discussion/conclusion section?	□
Why is this important?		No research study is perfect, and it is important that researchers are transparent about the limitations of their own work. For example, many study designs cannot provide causal evidence, and some inadvertent biases in the design can skew results. Other studies are based on more or less plausible assumptions. Such issues should be discussed either in the Discussion, or even in a dedicated Limitations section.	
Let’s dig deeper		Check for potential biases yourself. Here are some examples of potential sources of bias. 1. Check the study’s sample (methods section). Do the participants represent the target population? Testing a drug only on white male British smokers over 50 is probably not going to yield useful results for everyone living in the UK, for example. How many participants were there? There is no one-size-fits-all number of participants that makes a study good, but in general, the more participants, the stronger the evidence. 2. Was there a control group or control condition (e.g., placebo group or non-intervention condition)? If not, was there a reason? Having a control group helps to determine whether the treatment under investigation truly has an effect on an experimental group and reduces the possibility of making an erroneous conclusion. Not every study can have such controls though. Observational studies, for example, typically do not have a control group or condition, nor do case studies or reviews. If your preprint is on an observational study, case study, or review, this item may not apply. 3. Was there randomisation? That is, was the allocation of participants or groups of participants to experimental conditions done in a random way? If not, was there a reason? Randomisation is an excellent way to ensure that differences between treatment groups are due to treatment and not confounded by other factors. For example, if different treatments are given to patients based on their disease severity, and not at random, then the results could be due to either treatment effects or disease severity effects, or an interaction - we cannot know. However, some studies, like observational studies, case studies, or reviews, do not require randomisation. If your preprint is on an observational study, case study, or review, this item may not apply. 4. Was there blinding? Blinding means that some or all people involved in the study did not know how participants were assigned to experimental conditions. For example, if participants in a study do not know whether they are being administered a drug or a sham medication, the researchers can control for the placebo effect (people feeling better even after fake medication because of their expectation to get better). However, blinding is not always possible and cannot be applied in observational studies or reanalyses of existing non-blinded data, for example. If your preprint is on an observational study, case study, or review, this item may not apply). Other examples of sources of bias include spin, where results are reported in a misleading way to fit a story, and overinterpretation, where results are presented as if they mean more than they do.	

### PRECHECK: A checklist to evaluate preprints on COVID-19 and beyond

Preprints are manuscripts describing scientific studies that have not been peer-reviewed, that is, checked for quality by an unbiased group of scientists in the same field. Preprints are typically posted online on preprint servers (e.g. BioRxiv, MedRxiv, PsyRxiv) instead of scientific journals. Anyone can access and read preprints freely, but because they are not verified by the scientific community, they can be of lower quality, risking the spread of misinformation. When the COVID-19 pandemic started, a lack of understanding of preprints has led to low-quality research gaining popularity and even infiltrating public policy. Inspired by such events, we have created PRECHECK: a checklist to help *you* assess the quality of preprints in psychology and medicine, and decide their credibility. This checklist was created with scientifically literate non-specialists in mind, such as students of medicine and psychology, and science journalists.

The checklist contains 4 items. Read them and the *Why is this important?* Section underneath each of them. Check if the preprint you are reading fulfills the item’s criteria - if yes, tick the *Yes* box on the right. Generally, the more ticks on the checklist your preprint gets, the higher its quality, but this is only a superficial level of assessment. For a thorough, discriminative analysis of a preprint, please also consult the related *Let’s dig deeper* sections underneath each item. There are no tick boxes for this level of assessment, but you are welcome to make your own notes. When using the checklist, we recommend that you have both the preprint itself, and the webpage on the preprint server where the preprint was posted at hand. You can also check online whether the preprint has already been peer reviewed and published in a journal.

The checklist works best for studies with human subjects, using primary data (that the researchers collected themselves) or systematic reviews, meta-analyses and re-analyses of primary data. It is not ideally suited to simulation studies (where the data are computer-generated) or studies that have a qualitative part (e.g., like interview data, or other data that cannot be easily translated into numbers). In general, if the study sounds controversial, improbable, or too good to be true, we advise you to proceed with caution when reading the study.

## Discussion

Over four successive stages of refining the PRECHECK checklist, we arrived at a four-item checklist approved by internal and external review, that can help critically evaluate preprints. After a set of workshops with Bachelor students from Psychology and Medicine, and science journalists, we concluded that the checklist was in a state where it was ready to be used by scientifically literate non-specialists. Here we recapitulate the main findings and discuss their implications.

The results of the pilot testing by expert users can be divided into three key findings. First, across both tests, preprints deemed to be of high quality had consistently more positive responses on the checklist than preprints deemed to be of low quality. This indicates that the checklist might be effective at discriminating between preprints of high and low quality, since positive responses mean that the preprint in question ‘passes’ a given criterion for good quality. Importantly, this holds even when the checklist is reduced to only four items, which is important for maintaining the user-friendliness of the checklist.

That said, and we consider this the second key finding, the deep level seemed to be especially important for discriminating preprint quality. It was especially evident in the second pilot testing that combining the deep and superficial level is optimal for distinguishing high- from low-quality preprints. Combining both levels hence seems to provide a good starting set of aspects to search for and reflect upon when critically assessing a preprint of unknown quality. This is likely due to the nature of the questions at these two different levels of evaluation, as the superficial level probes surface level questions which can be considered a ‘bare minimum’ of quality. For example, it is standard scientific practice that the research question or aim of a study must be mentioned in its resulting manuscript, and indeed, most preprints, even ones that eventually end up being retracted, do offer this information. The issues with low-quality preprints rather seem to be in their designs, ethical considerations, handling of the data and interpretation of the results, ^22^ and how transparently they report their results. ^19^^,^^20^ In our checklist, the first set of issues is detected by the deep level of the Limitations item, as this level asks users to engage with the content of the research reported in the preprint, and to check for potential issues with the design. The second set of issues is addressed by the transparency and integrity question, at both the deep and superficial levels. Both levels have been successful at detecting problems in low-quality preprints, but also high-quality preprints, which brings us to the final key finding.

Third, for both high- and low-quality preprints, the checklist highlighted issues with transparency and research integrity. This mirrors the state of the COVID-19 literature, as we have seen that though the credibility of the research reported in preprints can be sound, ^37^^–^^39^ data and materials sharing may nonetheless often be lacking. ^19^^,^^20^ This could be taken as a weakness of the checklist, as this item is not particularly discriminative of preprint quality. However, we believe it is a strength, albeit an unintended one. On one hand, this feature highlights where there is room for improvement even in works that are otherwise of good quality. Thus, there is potential for integrating the checklist in peer-review procedures, as one of our external experts suggested. On the other hand, it informs non-specialist audiences of the importance of transparency and integrity when considering the quality of a manuscript. As another external expert pointed out, certain practices such as open data sharing are considered to be at the forefront of scientific endeavours, and not all researchers adhere to these standards, even if their research is sound. However, we believe that part of the reason for this non-adherence is that not everyone believes such Open Science practices to be sufficiently important, even despite a clear need to improve reproducibility in many areas of science. ^70^^,^^71^ By dedicating an entire item to issues of transparent reporting and research integrity, we hope to encourage non-specialists as well as scientists to both pay attention to and think critically about issues of transparency and integrity, in addition to the soundness of the research being reported.

Apart from the external expert comments already mentioned, the (online survey) review process revealed several important insights. It was illuminating that many experts agreed that the initial draft was too complex for non-scientist audiences, while there were also suggestions for additional items that required, in our view, expertise that non-scientists would not reasonably have. This situation made it clear that we had to define our target audience more precisely, and strike the right balance between simplicity and functionality in our checklist to make it both usable and accurate. The Preliminary implementation round confirmed that the checklist was indeed usable, as both our student and journalist cohorts across multiple sites appeared to understand how the checklist was supposed to be used during the workshop discussion. Further, in our workshops, the live surveys showed that there appeared to be an overlap in the students’ and journalists’ application of the checklist to the high- and low- quality preprints and our own. We do acknowledge that many important aspects of checking the quality of a scientific work that the experts mentioned were omitted, such as verifying the references, checking whether the work fills a gap in knowledge, and checking whether the statistical analyses are justified. Though this can be construed as a limitation, we believe it is justified by the feasibility constraint of the checklist being a teaching tool for audiences that will very likely not have the expertise to verify preprints in such ways.

Another prominent theme that emerged in the external experts’ comments was an implicit prioritization of randomized controlled trials, which could in turn disadvantage observational studies. Though we did not find the checklist to be as gravely biased, as all three of our high-quality preprints ^61^^–^^63^ were on observational studies, and they ‘passed’ the items on the checklist very well. We nonetheless appreciated the importance of this point, as much of the COVID-19 research reported in preprints was indeed observational. ^17^ In response, we made two substantial changes to the checklist. For one, we expanded the explanation of what observational studies are in the Why is this important? Section of Study type, by including concrete examples. This could help prevent non-experts from falsely identifying an observational study as having not indicated a study type, as unlike for randomized controlled trials, manuscripts on observational studies often do not explicitly mention that the research was observational. Moreover, we made it clear that the potential sources of bias mentioned in the ‘let’s dig deeper’ section in limitations were only examples, we added disclaimers for those sources of bias that may not apply to observational studies (control group/control condition, randomization, and blinding, specifically), and we included two more examples of biases that could apply to observational studies (spin and overinterpretation). We believe that these changes made the checklist more balanced towards all study types.

One important aspect of the checklist to note is its intended use as a teaching tool to guide non-specialist audiences to evaluate preprints more critically by giving them concrete aspects to search for in the manuscript at the superficial level, and food for thought that can lead to deeper insights at the deep level. The checklist is not meant to be a litmus test for whether a preprint is trustworthy or not, but rather a set of guidelines for users to make their own judgement, and a pedagogical tool to improve people’s competences and confidence at evaluating the quality of research. Though the superficial level alone could, in theory, be automated, as we have seen, the best results are obtained when the superficial and deep levels are combined, and it is the deep level that allows the user to delve deeper into issues of study design and potential biases. Nonetheless, it is useful to have a division into a superficial and deep level of assessment, as this allows greater flexibility for users to apply the checklist according to their needs, their experience, and their time constraints. For instance, we are aware that the 15-20 minutes of time that workshop participants were allocated may not be sufficient for everyone, especially individuals with no scientific training, to explore both levels. However, there is no one optimal setting or correct way to use the checklist for its use to be valid. Rather, it is meant to be flexible to suit the needs of various users, as long as it gets non-specialists engaged and thinking critically.

As a teaching tool meant to engage non-specialists, the current checklist could be a useful addition to existing approaches offering quick peer-review, such as Rapid Reviews\COVID-19. This specific initiative encourages short expert reviews, and in a particularly innovative approach, asks reviewers to use a Strength of Evidence Scale to select one of 5 options for how well the claims made in the preprint are supported by the data and method ( https://rrid.mitpress.mit.edu/guidelines). Thus, a fast, easy to interpret, and quantitative expert reviewer assessment is provided. Such assessments could be nicely complemented by the deep level aspects of the non-specialist “review” that our checklist can offer. For example, policymakers facing decisions based on preprints would be able to rely both on easy-to-understand quantitative expert assessments and qualitative non-specialist assessments for information.

## Limitations

The present study has several limitations that would have to be addressed in future research aiming for formal validation of the checklist. First, the selection of high- and low-quality preprints was based on proxies that may not objectively reflect a distinction between low- and high-quality studies. Moreover, the number of assessed preprints in both categories was at present too low for results to be assessed quantitatively, and to provide a formal validation of the checklist. For statistical validation to be possible, the number of preprints in each category would need to be much higher. A second limitation of the study is the limited and informal Preliminary implementation stage which was based on a population chosen mostly for convenience. At present, we did not collect any data from participants at this stage, as we did not have ethical approval to do so, and thus a formal and quantitative assessment could not be done. In order to conduct a formal statistical validation of the effectiveness of the checklist at this stage, a more careful recruitment and setup of an implementation stage would have been necessary. Finally, it is important to mention again that the checklist might not be applicable for all types of research. Our limited piloting suggests that it works best when applied to research with human subjects using primary data (or systematic reviews, meta-analyses and re-analyses of primary data).

## Conclusions

Over multiple iterative steps of internal and external review, pilot testing, and final polishing after the implementation phase, we created the PRECHECK checklist: a simple, user-friendly tool for helping scientifically literate non-experts critically evaluate preprint quality. We were inspired by the urgency of improving the public’s understanding of COVID-19 preprints, in which efforts to increase public awareness on preprint quality and competences at estimating preprint quality have been next to non-existent. Despite our COVID-19-related motivation, the PRECHECK checklist should be more broadly applicable to scientific works describing studies with human subjects.

## Authors' contributions (CRediT)

Conceptualization: LH, EV, EF; Data curation: NT, SS, RH; Formal Analysis: NT, SS, RH; Funding acquisition: LH, EV; Investigation: NT, SS, RH; Methodology: NT, SS, RH; Project administration: NT, SS, RH; Resources: LH, EV; Software: NT, SS, RH; Supervision: LH, EV, EF; Validation: NT, RH, SS, EF, EV, LH; Visualization: NT, SS, RH; Writing – original draft: NT, RH; Writing – review & editing: NT, RH, SS, EF, EV, LH.

## Data Availability

OSF: PRECHECK. https://doi.org/10.17605/OSF.IO/NK4TA. ^54^ This project contains the following underlying data:
• Code for Figure 1 folder. [R code in an RMD document to reproduce Figure 1 with the data that is also uploaded in this folder]. • Pilot testing Preprints folder. [high-quality subfolder containing the high-quality preprints chosen for the pilot testing (Bi *et al.,* - 2020 - Epidemiology and Transmission of COVID-19 in Shenz.pdf, Lavezzo *et al.,* - 2020 - Suppression of COVID-19 outbreak in the municipali.pdf, Wyllie *et al.,* - 2020 - Saliva is more sensitive for SARS-CoV-2 detection.pdf ), low-quality subfolder containing the low-quality preprints chosen for the pilot testing (Davido *et al.,* - 2020 - Hydroxychloroquine plus azithromycin a potential.pdf, Elgazzar *et al.,* - 2020 - Efficacy and Safety of Ivermectin for Treatment an.pdf, Pradhan *et al.,* 2020 - Uncanny similarity of unique inserts in the 2019-nCoV spike protein to HIV-1 gp120 and Gag.pdf ), and workshop_nonsense subfolder containing the preprint used for the fact-checking seminar (Oodendijk - 2020 - SARS-CoV-2 was Unexpectedly Deadlier than.pdf )] • Stage 1 folder. [Checklist Version after Stage 1 (20211018_PRECHECKchecklist.pdf ) and the pilot testing performed using that version of the checklist (Stage1_SensTest.xlsx)]. • Stage 2 folder [Checklist Version after Stage 2 (20220117_PRECHECKchecklist.pdf ), the form that was used to collect expert responses (ExpertSurveyForm.pdf ), and the replies to expert free text comments (Point-by-pointExpertReplies.pdf )] • Stage 3 folder [Checklist Version after Stage 3 (20220301_PRECHECKchecklist_afterComments.pdf ), the results of the sensitivity analyses done by the junior authors, NT (Stage3_SensTest_NT.xlsx) and RH (Stage3_SensTest_RH.xlsx)]. OSF: PRECHECK. https://doi.org/10.17605/OSF.IO/NK4TA. ^54^ This project contains the following extended data: In a subfolder entitled Materials for Manuscript:
•
20220520_FINAL_PRECHECKchecklist_afterComments_afterWorkshops.pdf. (Almost final version of the PRECHECK checklist). • 20231218_FINAL_PRECHECKchecklist_afterComments_afterWorkshops_afterReview.pdf. (Final version of the PRECHECK checklist). In a subfolder entitled Ethics:
• Ethics_checklist_PRECHECK.pdf (filled out University of Zurich ethical commission’s ethics review checklist) Data are available under the terms of the Creative Commons Zero “No rights reserved” data waiver (CC0 1.0 Public domain dedication). Repository: SRQR checklist for ‘Using an expert survey and user feedback to construct PRECHECK: A checklist to evaluate preprints on COVID-19 and beyond’. https://doi.org/10.17605/OSF.IO/JVHBW. ^72^

## Footnotes

^1^
For experience-based suggestions for teams of researchers interested in rapid peer-review, see Clyne and colleagues. ^40^

^2^
As we will explain below, high and low quality was decided based on proxies, and not on objective criteria, as we could not determine a set of such criteria that apply to all preprints across all disciplines that the checklist could possibly be used on. With this, when we refer to ‘high-quality’ and ‘low-quality’ preprints, we do so for brevity, and not because the preprints in question belong to predefined universally agreed upon categories of ‘good’ and ‘bad’. Further, the checklist is not a litmus test for whether a preprint is ‘good’ versus ‘bad’. Rather, it is intended to be used as a set of guidelines and a tool to get scientifically literate non-specialist to think critically about how they read preprints.
