# Supplementary material for: Using an expert survey and user feedback to construct PRECHECK: A checklist to evaluate preprints on COVID-19 and beyond
Source: F1000Res. 2024 Jun 3;12:588. Originally published 2023 Jun 1. [Version 3] doi: 10.12688/f1000research.129814.3 (PMC11231630; doi:10.12688/f1000research.129814.3)
Supplement: Peer review history

# Reviewer report: Reviewer response for version 3

**DOI:** 10.5256/f1000research.167306.r299409

**Published:** 2024-08-02

**Authors:** Adrian Lison

**Affiliations:** 1 ETH Zurich, Zürich, Zurich, Switzerland

The study by Turoman et al. presents a concise checklist for assessing the quality of health-related research papers and describes its multi-stage development process including the solicitation of expert opinions. Although the initial focus of the tool was on research related to COVID-19, the checklist developed is mostly generic and does not contain any COVID-19-specific items. The manuscript generally sets a positive example of extensive and open documentation of the research methodology. While no systematic validation of the checklist's ability to discriminate between low- and high-quality studies could be made, the authors discuss a preliminary application of the tool for teaching purposes. Below, I present a few remaining reservations and suggestions for improvement of the manuscript.

**Limitations w.r.t. observational studies**

 The limitations of the checklist with regard to observational studies are already discussed in detail in the manuscript. I would nevertheless like to raise one more point for discussion. The "dig deeper" sections of the final checklist now contain disclaimers that control groups and randomization may not apply to observational studies. While this is true in a strict sense, I think that a major criterion to distinguish high-quality and low-quality observational studies is how much effort was invested into ruling out potential sources of confounding and other biases. We have seen this extensively when reviewing observational studies assessing the effectiveness of non-pharmaceutical interventions during COVID-19 [1], where many studies derived causal interpretations from simple descriptive analyses without discussing potential sources of bias. On the other hand, high-quality studies invested major efforts to reduce confounding through study design (e.g. use of variation in intervention exposure between populations and over time) and specific methodologies (e.g. synthetic controls or difference-in-differences techniques). I am aware that such an assessment of the study methodology is probably to in-depth and technical for the proposed checklist. Still, I wonder if it could be stressed that even if observational studies do not have control groups in a strict sense, much can be done (or not done) to avoid bias.

**Development of first draft**

 While I generally appreciated the transparent documentation of the research methodology, I do have one point of critique: There are very few methodological details on how the first draft of the checklist was developed by the author team. As a response to an earlier comment by reviewer #1, a statement was added to the manuscript that "the first draft of the checklist was developed by the junior members of the team after an initial review of the literature and potential solutions (which forms the introduction of this paper)." I personally do not find this a sufficient explanation of the approach used to develop a first draft. Given the stepwise procedure of the study, I expect the first draft to have a strong influence on the final result. For example, even though the external experts were able to propose additional items, the review process seemed to be focused on the rating of existing items. It is thus relevant to know in more detail how the initial set was derived from the reviewed literature.

**Communication of the internal pilot testing**

 I think it is clear that the pilot testing conducted in this study does not constitute a validation of the checklist due to various points already raised by reviewer #2, including the limited number of preprints assessed, use of the same set of studies in Stage 1 and Stage 3, and, most importantly, the fact that the testing was conducted by the developers of the checklist themselves, and not by external experts. Personally, I think that the present study can be approved and is valuable even without independent validation. However, in the current version of the manuscript (version 3), I still see room for improvement w.r.t. communicating this limitation clearly to the reader.

 For example, the Results section of the abstract first mentions the external review (which was used to gather feedback on the checklist items) and then states that "when using both levels, the checklist was effective at discriminating a small set of high- and low-quality preprints" (which refers to the pilot testing). As a reader, I would thus by default assume that the pilot testing was also done by the external expert panel, when it was actually done by the study authors.

 Similarly, the second paragraph of the discussion starts with "The results of the pilot testing by expert users can be divided into three key findings." Again, I would recommend communicating more clearly that this refers to internal testing that was done by two members of the developer team.

**Educational resource vs. assessment tool**

 I figure from the discussion in the manuscript that the developed checklist is primarily meant as an educational resource/teaching tool. However, I wonder what the envisioned role of the PRECHECK project website is in this context. In its current form, the website appears like an assessment tool to me, and its description in the Introduction of the manuscript seems to reflect this, stating that "the aim was to develop a simple, user-friendly tool to guide non-scientists in their evaluation of preprint quality." I therefore wonder about the development team's plans going forward:

 If the PRECHECK project is primarily meant as an educational tool, then the website could communicate this aim more clearly and provide additional resources such as exemplary filled-out checklists for students, etc. If, in contrast, the goal is to offer an assessment tool, then it would be valuable to provide additional guidance and disclaimers as addressed in the discussion of the manuscript also on the website - in particular on the question of how to interpret the results of the checklist. Many potential issues could potentially be raised, for example, that bad quality studies should not be interpreted as evidence against the claims of the study. Also, further resources such as the quick peer-review projects mentioned in the manuscript could be linked. To summarize, I did not find the presentation of the project goals fully consistent, and think the PRECHECK website could be further optimized depending on what its primary aim is.

**The role of science journalism**

 I found it very convincing that (science) journalists could be a primary target group for the developed tool, since they operate in time- and resource-constrained environments during a public health emergency but their decisions to disseminate preprints in various media can have a substantial impact. The potential value of such checklists for journalism is a bit underdeveloped in the discussion and can be further highlighted, in my opinion.

**Minor points**

 Is it possible to improve the resolution of Figure 1?

 Introduction: "Nonetheless, a large portion of COVID-19 preprints show substantial changes in methods and results after peer-review." Are there specific references supporting this claim?

 Methods: "... workshop as homework; one group read,64 and the other group read)62". The formatting could be improved here.

 Checklist item 3a): "Is a protocol, study plan, or registration mentioned, and

 accessible (e.g., by hyperlink, by registration number)?" While I fully agree with the value of protocols and pre-registration, I think that this requirement might not be realistic for early preprints during a public health emergency. I still think it is a valuable item to include, but this limitation might be worth mentioning.

Is the work clearly and accurately presented and does it cite the current literature?

Partly

If applicable, is the statistical analysis and its interpretation appropriate?

Not applicable

Are all the source data underlying the results available to ensure full reproducibility?

Yes

Is the study design appropriate and is the work technically sound?

Yes

Are the conclusions drawn adequately supported by the results?

Yes

Are sufficient details of methods and analysis provided to allow replication by others?

Partly

Reviewer Expertise:

Infectious disease modeling, systematic literature review

I confirm that I have read this submission and believe that I have an appropriate level of expertise to confirm that it is of an acceptable scientific standard, however I have significant reservations, as outlined above.

## References

[1] : The methodologies to assess the effectiveness of non-pharmaceutical interventions during COVID-19: a systematic review. *Eur J Epidemiol* .2022;37(10) : 10.1007/s10654-022-00908-y 1003-1024 10.1007/s10654-022-00908-y 36152133 PMC9510554

# Reviewer report: Reviewer response for version 3

**DOI:** 10.5256/f1000research.167306.r285918

**Published:** 2024-07-08

**Authors:** Artemis Chaleplioglou

**Affiliations:** 1 University of West Attica, Aigaleo, Greece

I approve this version of this research article [version 3] as it is scientifically sound and interesting in preprints evaluation in general. The revisions by the authors are sufficient and undress well the reviewers’ concerns. The authors well addressed the importance of preprints in quick research dissemination, the scientific community response reflexes against preprint misleading cases, as well as other efforts in rapid evaluation of preprints. The PRECHECK project created by the authors represents an important initiative in evaluation of preprint quality by non-experts.

Is the work clearly and accurately presented and does it cite the current literature?

Partly

If applicable, is the statistical analysis and its interpretation appropriate?

Not applicable

Are all the source data underlying the results available to ensure full reproducibility?

Yes

Is the study design appropriate and is the work technically sound?

Yes

Are the conclusions drawn adequately supported by the results?

Yes

Are sufficient details of methods and analysis provided to allow replication by others?

Yes

Reviewer Expertise:

Library information science (LIS), Health information

I confirm that I have read this submission and believe that I have an appropriate level of expertise to confirm that it is of an acceptable scientific standard.

# Reviewer report: Reviewer response for version 2

**DOI:** 10.5256/f1000research.161594.r265330

**Published:** 2024-05-07

**Authors:** Somipam R. Shimray

**Affiliations:** 1 Babasaheb Bhimrao Ambedkar University, Lucknow, India

The authors provide a contemporary perspective on Covid-19, developing a checklist for non-experts to assess pre-prints. The authors used four stages of development to create the checklist: internal reviews and external expert reviews, final internal review and preliminary implementation stage. I will make a few brief observations. The manuscript could benefit from further improvement through the following questions and comments.

**Introduction**

- The inclusion of literature on the consequences of a preprint not meeting standard quality checks may be beneficial.
- The report lacks a comprehensive literature review, necessitating a thorough review to prevent duplication and resource wastage.

**Methods**

- The pilot testing consists of three high-quality and three low-quality preprints, which may be reasonable but not sufficient for validation.

**Results**

- Participants were given only 15-20 minutes to read preprints and complete a checklist, which is insufficient for novices or inexpert to understand and assess a scientific study manuscript. Thus, checklist's real-world usability is questioned, and more time should be allocated for manuscript assessment.
- The study's results require statistical validation.

**Discussion**

- This study’s key finding require statistical validation, as merely obtaining more positive responses ‘yes’ does not guarantee validation.
- The checklist's practical usability is questioned as it requires readers to consider 20 questions and points to evaluate a paper's quality.
- The authors created a user-friendly checklist to check quality of preprints. The checklist was a useful tool for educating prospective readers of medical research about peer review, publishing, preprints, evidence-based practice, research integrity, and transparency. This study aims to enhance research literacy among students, with the potential to be applied globally in various settings.
- These suggestions only improve the article; thus, I appreciate the authors considering them.

Is the work clearly and accurately presented and does it cite the current literature?

Partly

If applicable, is the statistical analysis and its interpretation appropriate?

Partly

Are all the source data underlying the results available to ensure full reproducibility?

Yes

Is the study design appropriate and is the work technically sound?

Yes

Are the conclusions drawn adequately supported by the results?

Yes

Are sufficient details of methods and analysis provided to allow replication by others?

Yes

Reviewer Expertise:

Research ethics

I confirm that I have read this submission and believe that I have an appropriate level of expertise to confirm that it is of an acceptable scientific standard, however I have significant reservations, as outlined above.

# Response

**Published:** 2024-05-30

**Authors:** Nora Turoman

**Affiliations:** University of Geneva, Switzerland

We thank the reviewer for their observations and recommendations.

 With the passage of time, any snapshot of the relevant literature that we can provide in the introduction to this manuscript will at some point become outdated, and thus a point for incoming reviews to point out. Thus, we can never provide a fully exhaustive literature review, especially while also respecting previous reviewer comments that our introduction was already too long to begin with. However, we believe that we have already discussed the consequences of preprint not meeting quality standards in the context of Covid-19 (which was the focus of our work) in the beginning of the introduction: “Even before the pandemic, concerns have been raised about the potential of such unvetted results and interpretation of findings leading to widespread misinformation. 10 Indeed, we have seen examples of non-peer-reviewed COVID-19-related claims 11 , 12 that were promptly uncovered as misleading or seriously flawed by the scientific community, 13 , 14 nonetheless infiltrate the public consciousness 15 and even public policy. 16”

 The methods comment has been addressed before in our response to the previous reviewers by adding a disclaimer in the limitation section: “First, the selection of high- and low-quality preprints was based on proxies that may not objectively reflect a distinction between low- and high-quality studies. Moreover, the number of assessed preprints in both categories would need to be much higher if results were to be assessed quantitatively.”. That said, we additionally qualified one of the sentences in the above paragraph. After addressing this and another one of your comments below, this part now reads: “Moreover, the number of assessed preprints in both categories was at present too low for results to be assessed quantitatively, and to provide a formal validation of the checklist. For statistical validation to be possible, the number of preprints in each category would need to be much higher.”

 Regarding the results, firstly, we have already addressed the issue of 15-20 minutes potentially not being enough time for every checklist user, in the text just before the Limitations section: “For instance, we are aware that the 15-20 minutes of time that workshop participants were allocated may not be sufficient for everyone to explore both levels. However, there is no one optimal setting or correct way to use the checklist for its use to be valid. Rather, it is meant to be flexible to suit the needs of various users, as long as it gets non-specialists engaged and thinking critically.” However, we have made this even clearer now by adding that “For instance, we are aware that the 15-20 minutes of time that workshop participants were allocated may not be sufficient for everyone, especially individuals with no scientific training, to explore both levels.”

 Secondly, regarding statistical analyses, we mentioned that we could not conduct any 1) at the stage of evaluating high- and low-quality preprints because, per the Pilot testing paragraph: “Due to the limited number of assessed preprints, only a qualitative summary of the results is possible, and no statistical analysis of these results is provided.”, and 2) in the Preliminary implementation stage, as we did not have ethical clearance to collect participant data (we mention throughout the manuscript that no data were collected in any of the workshops). To make these points clear, we have expanded the Limitations section, which now reads as follows: “First, the selection of high- and low-quality preprints was based on proxies that may not objectively reflect a distinction between low- and high-quality studies. Moreover, the number of assessed preprints in both categories was at present too low for results to be assessed quantitatively, and to provide a formal validation of the checklist. For statistical validation to be possible, the number of preprints in each category would need to be much higher. A second limitation of the study is the limited and informal Preliminary implementation stage which was based on a population chosen mostly for convenience. At present, we did not collect any data from participants at this stage, as we did not have ethical approval to do so, and thus a formal and quantitative assessment could not be done. In order to conduct a formal statistical validation of the effectiveness of the checklist at this stage, a more careful recruitment and setup of an implementation stage would have been necessary.”

 We thank you for your suggestions raised in your discussion points. We believe we have addressed them in the changes reported above.

# Reviewer report: Reviewer response for version 2

**DOI:** 10.5256/f1000research.161594.r265334

**Published:** 2024-04-26

**Authors:** Artemis Chaleplioglou

**Affiliations:** 1 University of West Attica, Aigaleo, Greece

During the COVID-19 pandemic the need for relative scientific information became imperative. The scientific journals pushed COVID-19 publications with fast-track reviewing and editing, whilst they released COVID-19 content freely available and reusable following their commitment on data sharing in public health emergencies (https://wellcome.org/press-release/sharing-research-data-and-findings-relevant-novel-coronavirus-ncov-outbreak and https://wellcome.org/press-release/publishers-make-coronavirus-covid-19-content-freely-available-and-reusable). Preprint repositories such as arXiv, SSRN, BioRxiv and MedRxiv jointly participated and signed the common statement of data sharing together with research fund organizations, universities, scientific societies, private companies, and scientific publishers, the day after the World Health Organization declared the COVID-19 outbreak as a public-health emergency of international concern. This statement ensured research finding availability via preprint servers before journal publication and to authors that preprints sharing ahead of submission wouldn’t pre-empt their publication in scientific journals. Under these conditions many investigators overcame any hesitations and contributed their findings including preliminary and interim works related to the pandemic to preprint repositories as elegantly demonstrated in Fig.1. It is important to refer to the collaborative participation of preprint repositories and scientific journals in the fast delivery of COVID-19 related data and information. In this report Turoman et al presents a checklist to qualitative evaluation of COVID-19 preprints. Rapid Reviews\COVID-19 ( https://rrid.mitpress.mit.edu/rrc19) initiative deserves to be mentioned as a platform reviewing COVID-19 preprints as well as because of its innovative approach summarized in its peer review guidelines ( https://rrid.mitpress.mit.edu/guidelines), and the delivery of the summary of reviews together with an easy to use and understanding by non-experts, strength of evidence scale key. This approach could be compared and discussed in parallel with the Turoman et al qualitative evaluation checklist.

Is the work clearly and accurately presented and does it cite the current literature?

Partly

If applicable, is the statistical analysis and its interpretation appropriate?

Not applicable

Are all the source data underlying the results available to ensure full reproducibility?

Yes

Is the study design appropriate and is the work technically sound?

Yes

Are the conclusions drawn adequately supported by the results?

Yes

Are sufficient details of methods and analysis provided to allow replication by others?

Yes

Reviewer Expertise:

Library information science (LIS), Health information

I confirm that I have read this submission and believe that I have an appropriate level of expertise to confirm that it is of an acceptable scientific standard, however I have significant reservations, as outlined above.

# Response

**Published:** 2024-05-30

**Authors:** Nora Turoman

**Affiliations:** University of Geneva, Switzerland

We thank you for your positive and insightful comment.

 We have now included the collaboration between journals and preprint repositories that you mentioned after the first reference to Figure 1: “Since the start of the pandemic, the number of preprints on COVID-19 has been steadily rising, with over 55,000 preprints to date (as of June 27, 2022; see also Figure 1). This proliferation can be explained by researchers hesitating less to upload their results as preprints, following the joint commitment to openly share COVID-19-related content by preprint servers, research funders, universities, scientific societies, private companies, and scientific publishers, the day after the World Health Organization declared the COVID-19 outbreak as a public-health emergency of international concern ( https://wellcome.org/press-release/sharing-research-data-and-findings-relevant-novel-coronavirus-ncov-outbreak; https://wellcome.org/press-release/publishers-make-coronavirus-covid-19-content-freely-available-and-reusable).”

 Rapid Reviews/COVID-19 has now been included in the list of existing approaches for offering quick peer review to Covid-19-related manuscripts: “Of the extant efforts to improve preprint quality, most have focused on introducing quality control via quick peer-review, e.g., Prereview ( https://www.prereview.org/), Review Commons ( https://www.reviewcommons.org/), PreLights ( https://prelights.biologists.com/), and Rapid Reviews/COVID-19 ( https://rrid.mitpress.mit.edu/rrc19).” We have also discussed how the checklist and the innovative Rapid Reviews/COVID-19 approach could co-exist in providing a more balanced set of information on preprints in the last paragraph before the Limitations section: “As a teaching tool meant to engage non-specialists, the current checklist could be a useful addition to existing approaches offering quick peer-review, such as Rapid Reviews\COVID-19. This specific initiative encourages short expert reviews, and in a particularly innovative approach, asks reviewers to use a Strength of Evidence Scale to select one of 5 options for how well the claims made in the preprint are supported by the data and method ( https://rrid.mitpress.mit.edu/guidelines). Thus, a fast, easy to interpret, and quantitative expert reviewer assessment is provided. Such assessments could be nicely complemented by the deep level aspects of the non-specialist “review” that our checklist can offer. For example, policymakers facing decisions based on preprints would be able to rely both on easy-to-understand quantitative expert assessments and qualitative non-specialist assessments for information.”

# Reviewer report: Reviewer response for version 1

**DOI:** 10.5256/f1000research.142526.r199813

**Published:** 2023-10-11

**Authors:** Alice Fleerackers

**Affiliations:** 1 Simon Fraser University, Burnaby, British Columbia, Canada

Thank you for giving me the opportunity to review this study. The topic is timely and relevant, and addresses a question I have spent a lot of time thinking about: How do you help people outside of academia vet the quality of preprint research?

 The study uses the DELPHI method to develop a checklist that ‘scientifically literate non-experts’ can use to assess the quality of preprint research. That is, the researchers developed an initial checklist, that was then iterated and refined through multiple rounds of expert feedback and testing. The experts who contributed feedback included academics (including those on the study team but also researchers with expertise in preprints, peer review, meta research, etc), as well as science journalists and students of medicine and psychology.

 The strengths of this study include the use of pre-defined protocols and quality reporting checklists (e.g., SRQR, Delphi method) and the choice to explicitly invite feedback from target users. I found that overall, the study was well-written. It provides links to iterations of the checklist, as well as data and protocols, which improves the transparency of the research.

 However, I have several concerns with the manuscript—some of which are major:

 First, I am very surprised that this did not require ethics review. While it does appear to fall outside of the scope of the university’s ethics guidelines, it was not clear from the manuscript whether participants were told their data would be used in a study. Rather students and journalists agreed to participate in a ‘workshop.’ Were they told their data would be used in reports/research? It would be helpful if the authors could clarity.

 Second, I have several concerns about the participants. While both science students and science journalists may be considered scientifically literate non-experts, these user groups have very different needs and likely very different understandings of academic publishing, research, and peer review—as well as different challenges and uses with respect to preprints. In addition, there are many other types of scientifically literate non-experts who could have been recruited to this study, such as policymakers, high school science teachers, ordinary people who studied science in the past, etc. A clear rationale for why you chose these two groups would considerably strengthen the manuscript.

 Relatedly, these groups (students, journalists) are incredibly diverse and there may be very different levels of science literacy within each group. For example, journalists in many beats use research in their reporting (not just those specialized in science), but some use it more frequently than others and some have professional training in a STEM field (e.g., they did a science degree before becoming journalists). Those who use research frequently, and/or have a research degree, likely have more in-depth knowledge than those who only occasionally use research or don’t have a STEM background. Similarly, some of the students in these two classes may have already completed a degree in STEM, or worked as researchers, while others may not have. Yet you provide no information about participants’ level of science literacy, previous experience with research, or education. Given that your target population is ‘scientifically literate non-experts’ then I would expect some assessment of science literacy, as well as a check that none of them are really ‘experts,’ to be a requirement when sampling participants. More broadly, it would be useful to know a bit more about both sets of participants (students and journalists), especially their educational backgrounds and level of experience with academic research, as this would provide a better understanding of who this checklist would be most useful for.

 Relatedly, very little information is provided about how the science journalists who were invited to participate in the workshops were recruited. How did you define ‘science journalist’? Did you include freelancers or staff reporters only? What about general news reporters who sometimes cover science? Did you only look at traditional, legacy journalism outlets or did you also consider newer forms of journalism? Providing more clarity around the inclusion or exclusion criteria for participating in the study, and the population of journalists from which you sampled, would improve the quality of the methods section.

 On a more minor note, you mention getting input on the checklist from “experts across four topic groups: research methodology (14 invitations), preprints (15 invitations), journal editors (15 invitations), and science journalists (10 invitations).” The rest of the paragraph explains who these people are in more detail, but at first reading it was not clear whether you were inviting researchers who study science journalists or the journalists themselves. Similarly, did you invite preprint server staff, preprint authors, or researchers who study preprints? I would adapt the language a bit here for clarity.  

 In addition, while I am not an expert in the DELPHI method, I do have some concerns about how it was implemented, given the conclusions that were drawn.

 First, this is a mixed method study, not a completely qualitative one, given that you gathered Likert-scale data (which was used to perform quantitative tests), as well as open-ended feedback. In addition, the Sensitivity Testing relied on binary data (i.e., yes/no) responses, which is quantitative. While I commend the authors for using Standards for Reporting Qualitative Research (SRQR), some standards for quantitative research have not been met. Specifically, there was no information about the degree to which the various users who used the checklist in the Sensitivity Testing agreed with one another. How often did one coder choose yes and the other choose no? Normally I would expect to see some form of intercoder reliability test here, especially if the goal of the study is to develop a checklist that can help people assess the quality of the research. If the checklist works well, then two individuals should be able to come to similar conclusions when using it. On a more minor note, in qualitative research, it is not true that “in general, the more participants, the stronger the evidence” (Turoman et al., 2023, p. 14). I would remove this statement or make it clear in the text that it relates specifically to the quantitative aspects of the research.

 Second, it strikes me that the checklist was never sensitivity tested by participants in the two target groups, only by members the study team. Why is that? Given that the research team are experts in this issue and had read and attempted to assess the quality of the checklist multiple times, they would likely be much more adept at applying the checklist than journalists or students using the checklist for the first time. This seems like a major limitation of the study, given that it seeks to provide a checklist that is useful for these groups. You do comment that “both our student and journalist cohorts across multiple sites appeared to understand how the checklist was supposed to be used” and that “there appeared to be an overlap in the students’ and journalists’ application of the checklist to the high- and low- quality preprints and our own.” (Turoman et al., 2023, p. 15), suggesting that you did examine the similarity in participants’ responses to the checklist questions. If this was the case, including the process used to make the comparison in the methods section, as well as the results of the comparison, would considerably strengthen the manuscript. If you did not compare the users’ responses in any systematic way, I do not think you can make conclusive claims such as, “The implementation round confirmed that the checklist was indeed usable (Turoman et al., 2023, p. 15) or . “Appearing” to understand how to use a checklist is not the same as understanding how to use it, nor using it in a way that is effective and reliable.

 Third, I found myself wondering about the choice of preprints used in the testing. How could reusing the same preprints over the various rounds have influenced results, especially given that the evaluation/application of the checklist was performed by the same two members each time (as mentioned above). Similarly, while the proxies you used to identify ‘high’ and ‘low’ quality preprints are creative, and has advantages over other proxy measures of quality (e.g., preprints published in high impact journals, or cited a lot), it seems like the Milestone List is based on the significance/usefulness of the research for addressing the COVID-19 crisis, not the integrity of the methods, data, study design, or reporting. Yet this is just a guess, because looking at the list, it's not clear at all what criteria were used to select the articles—it just says that " *Nature* highlighted key papers and preprints to help readers keep up with the flood of coronavirus research." What makes a paper "key" in this case? This seems very important given how central this list is to the method of the study. More broadly, I would appreciate a definition of "quality" somewhere earlier in this article, as well as a clear discussion of the limitations associated with the sample (e.g., limited to only three preprints, all of which were about COVID-19, and all of which are biomedical). The checklist seems very inappropriate for assessing the ‘quality’ of research or scholarship in other disciplines, such as the social sciences or humanities. You may want to rephrase the purpose and conclusions of the study to specify the focus on biomedical preprints, rather than on preprints in general.

 In addition, there is some literature missing from the review that seems worth citing with respect to journalists’ use of preprints, as well as the public’s understanding/reponses to media coverage of preprints. This includes some of my own work (I know, reviewers always do this), but also of other scholars. Here are some citations worth considering. I also encourage the authors to do an additional literature search, since more research may have come online recently of which I am not aware:

- van Schalkwyk, F., & Dudek, J. (2022). Reporting preprints in the media during the Covid-19 pandemic. *Public Understanding of Science*, *31*(5), 608–616. https://doi.org/10.1177/09636625221077392
- Massarani, L., & Neves, L. F. F. (2022). Reporting COVID-19 preprints: Fast science in newspapers in the United States, the United Kingdom and Brazil. *Ciência & Saúde Coletiva*, *27*, 957–968. https://doi.org/10.1590/1413-81232022273.20512021
- Massarani, L., Neves, L. F. F., Entradas, M., Lougheed, T., & Bauer, M. W. (2021). Perceptions of the impact of the COVID-19 pandemic on the work of science journalists: Global perspectives. *Journal of Science Communication*, *20*(07), A06. https://doi.org/10.22323/2.20070206
- Massarani, L., Neves, L. F. F., & Silva, C. M. da. (2021). Excesso e alta velocidade das informações científicas: Impactos da COVID-19 no trabalho de jornalistas. *E-Compós*. https://doi.org/10.30962/ec.2426
- Oliveira, T., Araujo, R. F., Cerqueira, R. C., & Pedri, P. (2021). Politização de controvérsias científicas pela mídia brasileira em tempos de pandemia: A circulação de preprints sobre Covid-19 e seus reflexos. *Revista Brasileira de História da Mídia*, *10*(1), Article 1. https://doi.org/10.26664/issn.2238-5126.101202111810
- Fleerackers, A., Moorhead, L. L., Maggio, L. A., Fagan, K., & Alperin, J. P. (2022). Science in motion: A qualitative analysis of journalists’ use and perception of preprints. *PLOS ONE*, *17*(11), e0277769. https://doi.org/10.1371/journal.pone.0277769
- Fleerackers, A., Riedlinger, M., Moorhead, L. L., Ahmed, R., & Alperin, J. P. (2022). Communicating scientific uncertainty in an age of COVID-19: An investigation into the use of preprints by digital media outlets. *Health Communication*, *37*(6), 726–738. https://doi.org/10.1080/10410236.2020.1864892
- Fleerackers, A., Shores, K., Chtena, N., & Alperin, J. P. (2023). *Unreviewed science in the news: The evolution of preprint media coverage from 2014-2021* (2023.07.10.548392). bioRxiv. https://doi.org/10.1101/2023.07.10.548392
- Ratcliff, C. L., Fleerackers, A., Wicke, R., Harvill, B., King, A. J., & Jensen, J. D. (2023). Framing COVID-19 preprint research as uncertain: A mixed-method study of public reactions. *Health Communication*, *0*(0), 1–14. https://doi.org/10.1080/10410236.2023.2164954
- Cyr, C., Cataldo, T. T., Brannon, B., Buhler, A., Faniel, I., Connaway, L. S., Valenza, J. K., Elrod, R., & Putnam, S. (2021). Backgrounds and behaviors: Which students successfully identify online resources in the face of container collapse. *First Monday*. https://doi.org/10.5210/fm.v26i3.10871
- Cataldo, T., Faniel, I., Buhler, A., Brannon, B., Connaway, L., & Putnam, S. (2023). Students’ Perceptions of Preprints Discovered in Google: A Window into Recognition And Evaluation. *College & Research Libraries*, *84*(1). https://doi.org/10.5860/crl.84.1.137
- Sebbah, B., Bousquet, F., & Cabanac, G. (2022). Le journalisme scientifique à l’épreuve de l’actualité « tout covid » et de la méthode scientifique. *Les Cahiers du journalisme*, *2*(8–9), R119–R135. https://doi.org/10.31188/CaJsm.2(8-9).2022.R119

Finally, I encourage the authors to make the implications and contributions of the work clearer. From a practical perspective, I found myself wondering, is this actually a checklist for vetting the quality of *preprints, *or for assessing research quality in general? Given that there are many, many checklists available for assessing the quality of peer reviewed research, answering this question seems very important. Perhaps the contribution is that this checklist could help nonexperts vet research—peer reviewed or not. I do not know this area well enough to know the conceptual, theoretical, or scholarly gaps that have been filled, but I am sure the authors can do so.

 I know that these are a lot of comments and concerns, but I have included them not to be rude or overly critical but because I believe addressing will make the study better able to contribute to an important issue that I care about deeply. I hope the authors find them helpful and are not discouraged from pursuing future research about journalists’ use of preprints. We need more of it!

Is the work clearly and accurately presented and does it cite the current literature?

Partly

If applicable, is the statistical analysis and its interpretation appropriate?

I cannot comment. A qualified statistician is required.

Are all the source data underlying the results available to ensure full reproducibility?

No

Is the study design appropriate and is the work technically sound?

Partly

Are the conclusions drawn adequately supported by the results?

Partly

Are sufficient details of methods and analysis provided to allow replication by others?

Partly

Reviewer Expertise:

Science journalism (specifically the use of preprints), science communication, health communication, preprints/open science

I confirm that I have read this submission and believe that I have an appropriate level of expertise to state that I do not consider it to be of an acceptable scientific standard, for reasons outlined above.

## References

[1] : Reporting preprints in the media during the COVID-19 pandemic. *Public Underst Sci* .2022;31(5) : 10.1177/09636625221077392 608-616 10.1177/09636625221077392 35196912 PMC9160779

[2] : Reporting COVID-19 preprints: fast science in newspapers in the United States, the United Kingdom and Brazil. *Cien Saude Colet* .2022;27(3) : 10.1590/1413-81232022273.20512021 957-968 10.1590/1413-81232022273.20512021 35293473

[3] : Perceptions of the impact of the COVID-19 pandemic on the work of science journalists: global perspectives. *Journal of Science Communication* .2021;20(07) : 10.22323/2.20070206 10.22323/2.20070206

[4] : Excesso e alta velocidade das informações científicas. *E-Compós* .2021; 10.30962/ec.2426 10.30962/ec.2426

[5] : Politização de controvérsias científicas pela mídia brasileira em tempos de pandemia: a circulação de preprints sobre Covid-19 e seus reflexos. *Revista Brasileira de História da Mídia* .2021;10(1) : 10.26664/issn.2238-5126.101202111810 10.26664/issn.2238-5126.101202111810

[6] : Science in motion: A qualitative analysis of journalists' use and perception of preprints. *PLoS One* .2022;17(11) : 10.1371/journal.pone.0277769 e0277769 10.1371/journal.pone.0277769 36409723 PMC9678308

[7] : Communicating Scientific Uncertainty in an Age of COVID-19: An Investigation into the Use of Preprints by Digital Media Outlets. *Health Commun* .2022;37(6) : 10.1080/10410236.2020.1864892 726-738 10.1080/10410236.2020.1864892 33390033

[8] : Unreviewed science in the news: The evolution of preprint media coverage from 2014-2021. *bioRxiv* .2023; 10.1101/2023.07.10.548392 10.1101/2023.07.10.548392

[9] : Framing COVID-19 Preprint Research as Uncertain: A Mixed-Method Study of Public Reactions. *Health Commun* .2023; 10.1080/10410236.2023.2164954 1-14 10.1080/10410236.2023.2164954 36683347

[10] : Backgrounds and behaviors: Which students successfully identify online resources in the face of container collapse. *First Monday* .2021; 10.5210/fm.v26i3.10871 10.5210/fm.v26i3.10871

[11] : Students’ Perceptions of Preprints Discovered in Google: A Window into Recognition And Evaluation. *College & Research Libraries* .2023;84(1) : 10.5860/crl.84.1.137 10.5860/crl.84.1.137

# Response

**Published:** 2024-01-18

**Authors:** Nora Turoman

**Affiliations:** University of Geneva, Switzerland

Point-by-point response to Reviewer 2 - Alice Fleerackers

 Thank you for giving me the opportunity to review this study. The topic is timely and relevant, and addresses a question I have spent a lot of time thinking about: How do you help people outside of academia vet the quality of preprint research?

 The study uses the DELPHI method to develop a checklist that ‘scientifically literate non-experts’ can use to assess the quality of preprint research. That is, the researchers developed an initial checklist, that was then iterated and refined through multiple rounds of expert feedback and testing. The experts who contributed feedback included academics (including those on the study team but also researchers with expertise in preprints, peer review, meta research, etc), as well as science journalists and students of medicine and psychology.

 The strengths of this study include the use of pre-defined protocols and quality reporting checklists (e.g., SRQR, Delphi method) and the choice to explicitly invite feedback from target users. I found that overall, the study was well-written. It provides links to iterations of the checklist, as well as data and protocols, which improves the transparency of the research.

 However, I have several concerns with the manuscript — some of which are major:

- First, I am very surprised that this did not require ethics review. While it does appear to fall outside of the scope of the university’s ethics guidelines, it was not clear from the manuscript whether participants were told their data would be used in a study. Rather students and journalists agreed to participate in a ‘workshop.’ Were they told their data would be used in reports/research? It would be helpful if the authors could clarity.

Reply: Thank you for your concern, we do care about transparency very much and paid attention to this aspect throughout the study. The situation was different for the student workshops and the journalist workshops. For the student workshops we write in the text and this corresponds to the actual process: “The responses were only analysed inasmuch as percentages of ‘yes’, ‘no’, and ‘partly/maybe’ responses were automatically generated by the respective platforms based on the responses entered by the students, during the class, for the purpose of in-class discussion. These responses were not recorded outside of this platform (e.g., on any project team member’s computer), nor were they subject to any formal statistical analyses performed on these responses, and the responses did not influence the content of the checklist in any way.” Therefore, the student workshops are simply described in this article to showcase how the PRECHECK checklist could be used in practice; the responses that the students gave as part of the workshop were not used to further inform the checklist. Hence, they were not told that their data would be used in a study because there was no intention to do so.

 For the journalist workshops, our intention was the same as above: we wanted to use the PRECHECK checklist purely for education, without gathering data for the project. But the journalists were informed through our presentation that we were the developers of the checklist, and some participants offered unsolicited feedback. In this situation it was clear to them that their feedback could be incorporated into a new version of the checklist and they did indeed give it for this purpose. In the article text we now write (addition from this revision in italics): “In the workshop for journalists, some participants offered unsolicited yet helpful feedback, which was incorporated into the finalised version of the checklist. *These participants were aware that their feedback would be used for this purpose through the presentation given during the workshop.*”

 Additionally we extended the section *Ethical considerations* with some more explanations of the facts.

  

- Second, I have several concerns about the participants. While both science students and science journalists may be considered scientifically literate non-experts, these user groups have very different needs and likely very different understandings of academic publishing, research, and peer review—as well as different challenges and uses with respect to preprints. In addition, there are many other types of scientifically literate non-experts who could have been recruited to this study, such as policymakers, high school science teachers, ordinary people who studied science in the past, etc. A clear rationale for why you chose these two groups would considerably strengthen the manuscript.

Reply: Thank you for this valid point. First we would like to mention that the section describing these workshops was situated in an incorrect location in the article. We have now moved it to the correct placement in the Methods Section.

We chose both groups first out of convenience, as we had direct access to them and an inherent interest in reaching them with our content. Initially our project was motivated by the Covid pandemic, during which results from preprints ended up as highlights in news outlets, and thus we wanted specifically to address science journalists. We are aware of the fact that Science journalists are actually a group of people who already have a lot of experience with vetting scientific literature. In contrast, our second group, the students, only have limited knowledge on the publishing system etc. Both the students at the University of Zurich and Geneva were at a stage in their studies where they needed to get used to using literature, the lectures in which our workshops were held had, in part, the purpose of introducing them to that. Consequently, given that they will have to read scientific papers as well as preprints for many of their future lectures and projects, we aimed at giving them a tool to help quality check what they plan on reading. We have added more explanations of these choices in the section *Study Design*. Specifically, we state “The chosen setup of the preliminary implementation phase does not allow a systematic validation of the checklist, especially since the target group was chosen mainly for convenience.”

 Relatedly, these groups (students, journalists) are incredibly diverse and there may be very different levels of science literacy within each group. For example, journalists in many beats use research in their reporting (not just those specialized in science), but some use it more frequently than others and some have professional training in a STEM field (e.g., they did a science degree before becoming journalists). Those who use research frequently, and/or have a research degree, likely have more in-depth knowledge than those who only occasionally use research or don’t have a STEM background. Similarly, some of the students in these two classes may have already completed a degree in STEM, or worked as researchers, while others may not have. Yet you provide no information about participants’ level of science literacy, previous experience with research, or education. Given that your target population is ‘scientifically literate non-experts’ then I would expect some assessment of science literacy, as well as a check that none of them are really ‘experts,’ to be a requirement when sampling participants. More broadly, it would be useful to know a bit more about both sets of participants (students and journalists), especially their educational backgrounds and level of experience with academic research, as this would provide a better understanding of who this checklist would be most useful for.

Reply:  We understand why this information could be of interest, but since we did not collect any data on the workshop participants we cannot provide this information. The text states that “The responses were only analysed inasmuch as percentages of ‘yes’, ‘no’, and ‘partly/maybe’ responses were automatically generated by the respective platforms based on the responses entered by the students, during the class, for the purpose of in-class discussion. These responses were not recorded outside of this platform (e.g., on any project team member’s computer), nor were they subject to any formal statistical analyses performed on these responses, and the responses did not influence the content of the checklist in any way. We did not ask any question about the workshop participants’ educational background or level of experience with academic research.” We thus added the issues raised here to the new Limitations section of the manuscript.

 Relatedly, very little information is provided about how the science journalists who were invited to participate in the workshops were recruited. How did you define ‘science journalist’? Did you include freelancers or staff reporters only? What about general news reporters who sometimes cover science? Did you only look at traditional, legacy journalism outlets or did you also consider newer forms of journalism? Providing more clarity around the inclusion or exclusion criteria for participating in the study, and the population of journalists from which you sampled, would improve the quality of the methods section.

Reply: Thank you for pointing this out, we now tried to be clearer in the text. The first workshop for science journalists was organised for science journalists employed at the Universities of Geneva (i.e. the members of the respective Communication Departments). The parenthesis has been added to the text during this revision and explains that we again used a “convenience sample” within our own institutions. Second, we were invited to present at a workshop for science journalists organised by a third party, the Swiss Association of Science Journalists, for which the recruitment was not in our hands. As explained on the association’s website, ordinary members of the association must attest that they work as journalists for an independent media outlet and their work should primarily focus on scientific, medical, technical or science policy issues.

 We would like to stress again that none of the workshops were intended to collect data for a validation of the checklist. All events were, what we now call, preliminary implementation events, where we showcased the checklist for use in a teaching setting.

 On a more minor note, you mention getting input on the checklist from “experts across four topic groups: research methodology (14 invitations), preprints (15 invitations), journal editors (15 invitations), and science journalists (10 invitations).” The rest of the paragraph explains who these people are in more detail, but at first reading it was not clear whether you were inviting researchers who study science journalists or the journalists themselves. Similarly, did you invite preprint server staff, preprint authors, or researchers who study preprints? I would adapt the language a bit here for clarity. 

Reply: Thanks a lot for this comment which shows that more information is needed. We realised the sentence you also copied presenting the four topics was confusing, since we used jobs (editors and journalists) to refer to topics. Hence, this was changed now to *journal publishing (15 invitations), and science journalism (10 invitations)*. We also added another clarifying sentence: *Thus, we invited science journalists, journal editors, as well as researchers whose research focuses on research methodology and preprints.*

 In addition, while I am not an expert in the DELPHI method, I do have some concerns about how it was implemented, given the conclusions that were drawn.

 First, this is a mixed method study, not a completely qualitative one, given that you gathered Likert-scale data (which was used to perform quantitative tests), as well as open-ended feedback. In addition, the Sensitivity Testing relied on binary data (i.e., yes/no) responses, which is quantitative. While I commend the authors for using Standards for Reporting Qualitative Research (SRQR), some standards for quantitative research have not been met. Specifically, there was no information about the degree to which the various users who used the checklist in the Sensitivity Testing agreed with one another. How often did one coder choose yes and the other choose no? Normally I would expect to see some form of intercoder reliability test here, especially if the goal of the study is to develop a checklist that can help people assess the quality of the research. If the checklist works well, then two individuals should be able to come to similar conclusions when using it. On a more minor note, in qualitative research, it is not true that “in general, the more participants, the stronger the evidence” (Turoman et al., 2023, p. 14). I would remove this statement or make it clear in the text that it relates specifically to the quantitative aspects of the research.

Reply: Thank you for this diligent point. It is true that we quantitatively assessed the responses from the expert panel. But this is the only real quantitative aspect of this study. The sensitivity testing, now called pilot testing, did not quantitatively assess the procedure. We added the following sentence to the text: *Due to the limited number of assessed preprints only a qualitative summary of the results is possible and no statistical analysis of these results is provided.*

 You are completely correct that the remark in the checklist “There is no one-size-fits-all number of participants that makes a study good, but in general, the more participants, the stronger the evidence.” of course only applies to quantitative studies. But the checklist is preceded by the disclaimer “The checklist works best for studies with human subjects, using primary data (that the researchers collected themselves) or systematic reviews, meta-analyses and re-analyses of primary data. It is not ideally suited to simulation studies (where the data are computer-generated).”, which we have now expanded to state the following in the last sentence: “It is not ideally suited to simulation studies (where the data are computer-generated) or studies that have a qualitative part (e.g., like interview data, or other data that cannot be easily translated into numbers).” We have realised that the disclaimer had not initially made it into the manuscript, only the document in OSF itself, and the web version of the checklist. Thus, we have added the disclaimer into the manuscript now (see Table 2), and the updated version on the OSF.

 Second, it strikes me that the checklist was never sensitivity tested by participants in the two target groups, only by members of the study team. Why is that? Given that the research team are experts in this issue and had read and attempted to assess the quality of the checklist multiple times, they would likely be much more adept at applying the checklist than journalists or students using the checklist for the first time. This seems like a major limitation of the study, given that it seeks to provide a checklist that is useful for these groups. You do comment that “both our student and journalist cohorts across multiple sites appeared to understand how the checklist was supposed to be used” and that “there appeared to be an overlap in the students’ and journalists’ application of the checklist to the high- and low- quality preprints and our own.” (Turoman et al., 2023, p. 15), suggesting that you did examine the similarity in participants’ responses to the checklist questions. If this was the case, including the process used to make the comparison in the methods section, as well as the results of the comparison, would considerably strengthen the manuscript. If you did not compare the users’ responses in any systematic way, I do not think you can make conclusive claims such as, “The implementation round confirmed that the checklist was indeed usable (Turoman et al., 2023, p. 15) or . “Appearing” to understand how to use a checklist is not the same as understanding how to use it, nor using it in a way that is effective and reliable.

Reply: Again, thank you for this valid point. We made sure that the distinction between expert testers in the pilot testing phases (formerly sensitivity testing) is made more clearly, e.g. in the abstract with the adapted sentence “When using both levels of evaluation, the checklist was effective for expert users at discriminating high- from low-quality preprints in a small set of example preprints.” During the workshops we used the classroom response systems (Votamatic and Klicker) provided by our universities to discuss with the audience what we believe are the correct answers to the checklist questions for two specific preprints that we also included in the pilot testing phase. This workshop discussion phase is the reason why we mention that participants appeared to understand the use of the checklist and provided in majority the same answers as ourselves. We adapted the wording of this section as follows: *The preliminary implementation round confirmed that the checklist was indeed usable, as, during the workshop discussion, both our student and journalist cohorts across multiple sites appeared to understand how the checklist was supposed to be used. Further, in our workshops, the live surveys showed that there appeared to be an overlap in the students’ and journalists’ application of the checklist to the high- and low- quality preprints and our own.*

 Third, I found myself wondering about the choice of preprints used in the testing. How could reusing the same preprints over the various rounds have influenced results, especially given that the evaluation/application of the checklist was performed by the same two members each time (as mentioned above). Similarly, while the proxies you used to identify ‘high’ and ‘low’ quality preprints are creative, and has advantages over other proxy measures of quality (e.g., preprints published in high impact journals, or cited a lot), it seems like the Milestone List is based on the significance/usefulness of the research for addressing the COVID-19 crisis, not the integrity of the methods, data, study design, or reporting. Yet this is just a guess, because looking at the list, it's not clear at all what criteria were used to select the articles—it just says that "Nature highlighted key papers and preprints to help readers keep up with the flood of coronavirus research." What makes a paper "key" in this case? This seems very important given how central this list is to the method of the study. More broadly, I would appreciate a definition of "quality" somewhere earlier in this article, as well as a clear discussion of the limitations associated with the sample (e.g., limited to only three preprints, all of which were about COVID-19, and all of which are biomedical). The checklist seems very inappropriate for assessing the ‘quality’ of research or scholarship in other disciplines, such as the social sciences or humanities. You may want to rephrase the purpose and conclusions of the study to specify the focus on biomedical preprints, rather than on preprints in general.

Reply:  Thank you very much for these questions, which in part were also asked by Reviewer 1. In the new Limitations sections we discuss the limitations of the selection of the sample of preprints. We also warn in the Pilot testing section: *Due to the limited number of assessed preprints, only a qualitative summary of the results is possible and no statistical analysis of these results is provided. *As a consequence the set of chosen preprints is, in our opinion, not completely central to the presented project and we chose not to comment any further on the process of how papers on the Milestone list were selected.

 We also refrained from defining “quality” of a preprint or a publication in general, as already mentioned in the manuscript (Footnote 2): *As we will explain below, high and low quality was decided based on proxies, and not on objective criteria, as we could not determine a set of such criteria that apply to all preprints across all disciplines that the checklist could possibly be used on. With this, when we refer to ‘high-quality’ and ‘low-quality’ preprints, we do so for brevity, and not because the preprints in question belong to predefined universally agreed upon categories of ‘good’ and ‘bad’. *Here, as in response to reviewer 1, we use retraction as an imperfect proxy of low quality.

 Finally, we did adapt our conclusion to be narrower: *Despite our COVID-19-related motivation, the PRECHECK checklist should be more broadly applicable to scientific works describing biomedical studies with human subjects.*

 In addition, there is some literature missing from the review that seems worth citing with respect to journalists’ use of preprints, as well as the public’s understanding/reponses to media coverage of preprints. This includes some of my own work (I know, reviewers always do this), but also of other scholars. Here are some citations worth considering. I also encourage the authors to do an additional literature search, since more research may have come online recently of which I am not aware:

 [LIST OF REFERENCES]

Reply: Thanks a lot for pointing us to further literature we were unaware of. Some of those papers were published after our first submission. We reviewed the papers for their relevance for our manuscript and added many of them in our introduction/literature review.

 Finally, I encourage the authors to make the implications and contributions of the work clearer. From a practical perspective, I found myself wondering, is this actually a checklist for vetting the quality of preprints, or for assessing research quality in general? Given that there are many, many checklists available for assessing the quality of peer reviewed research, answering this question seems very important. Perhaps the contribution is that this checklist could help nonexperts vet research—peer reviewed or not. I do not know this area well enough to know the conceptual, theoretical, or scholarly gaps that have been filled, but I am sure the authors can do so.

Reply: We repeatedly refer to our checklist as a “teaching tool”, as for example in this sentence in the discussion: *One important aspect of the checklist to note is its intended use as a teaching tool to guide non-specialist audiences to evaluate preprints more critically by giving them concrete aspects to search for in the manuscript*. We additionally decided to delete the last sentence in our Conclusions section ( *With this, and the fact that our target audience could use the checklist, we believe that the checklist has great potential to help guide non-scientists’ understanding of scientific content, especially in preprint form.*) because this claim is not actually supported by our project.

 I know that these are a lot of comments and concerns, but I have included them not to be rude or overly critical but because I believe addressing will make the study better able to contribute to an important issue that I care about deeply. I hope the authors find them helpful and are not discouraged from pursuing future research about journalists’ use of preprints. We need more of it!

Reply: We greatly appreciate your comments and were able to integrate them in our manuscript. We are convinced that the changes we made thanks to your comments improved the clarity and relevance of our manuscript.

# Reviewer report: Reviewer response for version 1

**DOI:** 10.5256/f1000research.142526.r176427

**Published:** 2023-07-18

**Authors:** Paul Glasziou, Oyungerel Byambasuren

**Affiliations:** 1 Institute for Evidence-Based Healthcare, Bond University, Robina, Queensland, Australia

Turoman and colleagues have reported on the development of a much-needed assessment tool to help lay people to check preprint quality. The rationale for the study were clearly laid out. They went through four steps of development to create the checklist: initial internal and external reviews, final internal review and implementation. The resulting checklist has 4-categories – research question, study type, transparency and integrity, and limitations. Their reporting was well done following the Standards for Reporting Qualitative Research.

 This study aimed to develop a brief checklist to guide non-experts in assessing pre-prints on Covid-19, but clearly can be extended to other types of research. The authors should be congratulated on making an interesting attempt. It represents a good first step, but I have a number of reservations about their validation process and the current checklist.

 There are a few questions and comments below that could further improve this manuscript.

**Introduction**: could be more concise particularly paragraph 1 and 3.

**Methods**:

- Ethics clearance was not required. The study has a repository on Open Science Framework with all relevant documents, but it’s not clear whether it was submitted *a priori*. Please clarify.
- The researchers have not reported whether they conducted a literature review before developing their first draft of the checklist with six items. Checking existing literature in the beginning of research helps reduce research waste and needless duplication or repetition. Please clarify and if not conducted justify this omission.
- The sensitivity testing is very limited using only three "high-quality" and three "low quality" preprints assessed with the checklist. That might be reasonable pilot testing, but far from a sensitivity testing or validation.
- Whilst choosing major COVID-19-related research as “high” quality test papers for the sensitivity analysis was understandable, choosing retracted preprints for the “low” quality set is not necessarily appropriate. Manuscripts could be of ‘good’ quality methodologically, yet they could be retracted due to data fabrication or irregularities. So, the fact that they were retracted doesn’t automatically mean they were of “low” quality. Please add it to the limitations.

**Results:**

- Second iteration of the checklist was trialled with psychology and medicine students and science journalists. They were given 15-20 minutes to read the preprints and complete the checklist as a group. For inexperienced people, 15-20 minutes is not enough time to understand a scientific study manuscript let alone assess the quality of it. This raises a concern about the real-world usability of this checklist.
- Sensitivity tests of the checklist drafts are not statistically analysed. Yes and no answers were compared between testers and workshop participants and between ‘high’ and ‘low’ quality preprints to make a final conclusion.  
- Final draft checklist was again tested with students and journalists. It now has 4 categories: research questions, study type, transparency and integrity, and limitations. Each category has a brief statement explaining why this category is important and the latter 3 categories each have 4-5 additional questions to evaluate the manuscript in “a deeper level”.
- The authors note that the checklist is only applicable to research in humans, and not for in vitro or animal studies. Implicitly then this is in the first check item that needs to be added to their checklist

**Discussion**: The authors claim that there are 3 key findings from this study:

- They state that this checklist is **effective in discerning between high- and low-quality studies**, because they observed that ‘high’ quality studies get more ‘yes’ answers than the ‘low’ quality ones. Is this claim warranted without proper statistical analysis?
- They continue that the ‘deep level’ questions are especially important in discriminating the quality of preprints. This means the reader must consider about 20 different questions and points to make a quality judgement of a paper, which brings us to the issue of practical usability of this checklist. To make a reasonable judgement, a reader will need to read the preprint more than once to be able to orient them and to find relevant information pertaining to the checklist. This would take more than 20 minutes the authors allowed the workshop participants. Please elaborate on why a reader should spend that much time to use this checklist themselves over a social media post by an ‘expert’ dissecting the paper, for example.
- Third key finding is that this checklist highlighted issues with transparency and research integrity. We agree that any quality assessment attempt should consider these issues and this checklist could help improve the understanding and literacy of lay people regarding issues around research integrity and transparency.

Overall, the authors had their work cut out in attempting to create “a simple and user-friendly tool” to check quality of preprints for educated lay people as it requires a fine balance between usability and accuracy. The resulting checklist was shown to be a useful tool for educating potential readers of medical research on wider issues of publishing, preprints, peer review, evidence-based practice, research integrity, and transparency. This study sparks important research on improving research literacy of lay people and we hope that this study would be extended and applied to many different settings around the world.

 Given the limitations I would suggest the authors need to be more circumspect In their conclusions about the usefulness of this current checklist given the extremely limited validity checking of it this is a very useful pilot but I would say I was only very preliminary work.

 Minor. In figure 2 the "implementation stage" might better be labelled a "piloting stage" - this was really pilot testing the draft checklist out on a number of groups before finalising the checklist, rather than implementation to the wider community.

Is the work clearly and accurately presented and does it cite the current literature?

Yes

If applicable, is the statistical analysis and its interpretation appropriate?

Not applicable

Are all the source data underlying the results available to ensure full reproducibility?

Yes

Is the study design appropriate and is the work technically sound?

Yes

Are the conclusions drawn adequately supported by the results?

Partly

Are sufficient details of methods and analysis provided to allow replication by others?

Yes

Reviewer Expertise:

Evidence-based practice, overdiagnosis, non-drug interventions,

We confirm that we have read this submission and believe that we have an appropriate level of expertise to confirm that it is of an acceptable scientific standard, however we have significant reservations, as outlined above.

# Response

**Published:** 2024-01-18

**Authors:** Nora Turoman

**Affiliations:** University of Geneva, Switzerland

Point-by-point responses to Reviewer 1 - Paul P. Glasziou and Oyungerel Byambasuren

 Turoman and colleagues have reported on the development of a much-needed assessment tool to help lay people to check preprint quality. The rationale for the study were clearly laid out. They went through four steps of development to create the checklist: initial internal and external reviews, final internal review and implementation. The resulting checklist has 4-categories – research question, study type, transparency and integrity, and limitations. Their reporting was well done following the Standards for Reporting Qualitative Research.

 This study aimed to develop a brief checklist to guide non-experts in assessing pre-prints on Covid-19, but clearly can be extended to other types of research. The authors should be congratulated on making an interesting attempt. It represents a good first step, but I have a number of reservations about their validation process and the current checklist.

 There are a few questions and comments below that could further improve this manuscript.

**Introduction**: could be more concise particularly paragraph 1 and 3.

Reply: Paragraphs 1 and 3 were shortened, while we also added additional literature in the introduction as requested by the second reviewer (Alice Fleerackers).

**Methods**:

- Ethics clearance was not required. The study has a repository on Open Science Framework with all relevant documents, but it’s not clear whether it was submitted a priori. Please clarify.

Reply: No application for ethics clearance was submitted a priori. As we state in the text  in the section *Ethical considerations* the verification was post-hoc: “A post-hoc verification with the University of Zurich ethical commission’s ethics review checklist (available in in the OSF repository for this project) confirmed that we did not require ethical approval for the current project per the regulations of the University of Zurich.” Unfortunately, we only realised during this revision that the document was provided to the editorial office but was not put on the OSF repository. This has been rectified now and the document is available here: https://osf.io/uysze. Additionally we extended the section *Ethical considerations* with additional explanations.

  

- The researchers have not reported whether they conducted a literature review before developing their first draft of the checklist with six items. Checking existing literature in the beginning of research helps reduce research waste and needless duplication or repetition. Please clarify and if not conducted justify this omission.

Reply: In order to draft the first version of the checklist, a literature review was performed to understand the state of disseminating COVID-19-related results via preprints at the time, the impact of such dissemination, and what was already present and what was missing in terms of mitigating some negative impacts of such dissemination. The results of this literature review enter in the introduction of the manuscript. In the description of the study design we added that the first draft of the checklist was *developed by the junior members of the team after an initial review of the literature and potential solutions (which forms the introduction of this paper)*.

  

- The sensitivity testing is very limited using only three "high-quality" and three "low quality" preprints assessed with the checklist. That might be reasonable pilot testing, but far from a sensitivity testing or validation.

Reply: You are making a valid point. We adapted the wording and changed the term “sensitivity testing” to “pilot testing”. As explained later, we also extended the list of limitations of our work and the issue of limited testing is now explicitly mentioned.

  

- Whilst choosing major COVID-19-related research as “high” quality test papers for the sensitivity analysis was understandable, choosing retracted preprints for the “low” quality set is not necessarily appropriate. Manuscripts could be of ‘good’ quality methodologically, yet they could be retracted due to data fabrication or irregularities. So, the fact that they were retracted doesn’t automatically mean they were of “low” quality. Please add it to the limitations.

Reply: Again, you raise a valid point. Our initial reasoning was that if the preprint authors, for example, fabricate their data, they might also not transparently disclose other aspects (limitations, conflict of interests, data sharing), so that our checklist could identify the preprint as being of poor quality. As such, retraction is used here as an imperfect proxy of low quality. We already discussed the difficulty of the selection of appropriate “low” quality preprints, but mention it now as a true limitation of our study in a dedicated Limitations section and in an amended sentence in the Pilot testing section “[...] with retraction being an imperfect but feasible proxy for low quality [...]”.

**Results**:

- Second iteration of the checklist was trialled with psychology and medicine students and science journalists. They were given 15-20 minutes to read the preprints and complete the checklist as a group. For inexperienced people, 15-20 minutes is not enough time to understand a scientific study manuscript let alone assess the quality of it. This raises a concern about the real-world usability of this checklist.

Reply: Thank you for raising this point. First we would like to mention that the section describing these workshops was situated in an incorrect location in the article. We have now moved it to the correct placement in the Methods Section.

In fact, we designed the checklist to be used as a quick tool, with the option of digging deeper depending on interest, background, and preference. Hence, 15-20 minutes should be enough to decide whether it is worth spending more time reading a preprint. As such, the superficial level of our checklist provides a starting point of concrete aspects to search for without a deep understanding of the underlying science. The deeper level allows a more in-depth consideration, and for some aspects at this level, it is true that one needs to understand the presented study more thoroughly, which would take longer. That said, the less experienced student audience was given the preprints in advance and had to read at least one of them as homework (see Pilot implementation phase). In light of this comment, we have now added more detail in the Discussion section.

  

- Sensitivity tests of the checklist drafts are not statistically analysed. Yes and no answers were compared between testers and workshop participants and between ‘high’ and ‘low’ quality preprints to make a final conclusion. 

Reply: No, there were no statistical tests and analyses produced, and this was never intended. During the workshops we did not even collect data due to ethical considerations. This sensitivity (or pilot) testing should therefore be regarded as exploratory; we added a sentence to the corresponding section in the Methods section: “There were no formal statistical analyses performed on these responses, and the responses did not influence the content of the checklist in any way.”

  

- Final draft checklist was again tested with students and journalists. It now has 4 categories: research questions, study type, transparency and integrity, and limitations. Each category has a brief statement explaining why this category is important and the latter 3 categories each have 4-5 additional questions to evaluate the manuscript in “a deeper level”.
- The authors note that the checklist is only applicable to research in humans, and not for in vitro or animal studies. Implicitly then this is in the first check item that needs to be added to their checklist.

Reply: We understand your point. There is already a disclaimer on the checklist stating that it is mainly applicable for research in humans. We realised that the disclaimer was not added to the manuscript, and we have added it now (see Table 2). We believe that this is a better solution than adding an item to the checklist which could be wrongly understood as an additional quality criterion. We also discuss this in the new Limitations section.

**Discussion**: The authors claim that there are 3 key findings from this study:

- They state that this checklist is effective in discerning between high- and low-quality studies, because they observed that ‘high’ quality studies get more ‘yes’ answers than the ‘low’ quality ones. Is this claim warranted without proper statistical analysis?

Reply: Thank you for this very good point. As below, we changed to more cautious wording in the Abstract and the Results section, and we added an explanatory sentence in the Methods section:

 “When using both levels of evaluation, the checklist was effective for expert users at discriminating high- from low-quality preprints in a small set of example preprints.”

 “This indicates that the checklist might be effective at discriminating between preprints of high and low quality.”

“Due to the limited number of assessed preprints, only a qualitative summary of the results is possible and no statistical analysis of these results is provided..”

  

- They continue that the ‘deep level’ questions are especially important in discriminating the quality of preprints. This means the reader must consider about 20 different questions and points to make a quality judgement of a paper, which brings us to the issue of practical usability of this checklist. To make a reasonable judgement, a reader will need to read the preprint more than once to be able to orient them and to find relevant information pertaining to the checklist. This would take more than 20 minutes the authors allowed the workshop participants. Please elaborate on why a reader should spend that much time to use this checklist themselves over a social media post by an ‘expert’ dissecting the paper, for example.

Reply: The workshops we organised are only one example of how to use the checklist in guiding non-experts in critical assessment. Their purpose was to teach university students and science journalists how the checklist works, in a way that blends in with their work schedules, and not to impose an ideal setting or time limit on their use of the checklist. Indeed, the superficial level lends itself to a rather quick check by searching digitally in the pdf of the preprint. For inexperienced users of the scientific literature, this provides a way of knowing how to start such an assessment. For more experienced users, e.g. the science journalists, the deeper level provides a good starting set of aspects to reflect upon. In a different setting from our workshops, one could start with the superficial level, and choose one specific theme from the deeper level to be discussed, e.g. bias. Depending on the user’s experience and level of involvement, this or any use of the ‘deep level’ could take various amounts of time. Again, we must stress that the checklist is primarily a way to get non-specialists thinking about how to assess preprint quality for themselves - not every question must be considered for the exercise to be valid, as long as it gets non-specialists engaged and thinking critically. We have now clarified this in the Discussion section.

If the checklist is used on its own, and specifically in relation to a social media post, it provides, in our opinion, a way to check that the post did indeed consider important aspects of preprint quality (which are summarised in the checklist). If the answers to all deeper level questions can be found in the post, 20 minutes could again be sufficient time to dig deeper.

  

- Third key finding is that this checklist highlighted issues with transparency and research integrity. We agree that any quality assessment attempt should consider these issues and this checklist could help improve the understanding and literacy of lay people regarding issues around research integrity and transparency.

Overall, the authors had their work cut out in attempting to create “a simple and user-friendly tool” to check quality of preprints for educated lay people as it requires a fine balance between usability and accuracy. The resulting checklist was shown to be a useful tool for educating potential readers of medical research on wider issues of publishing, preprints, peer review, evidence-based practice, research integrity, and transparency. This study sparks important research on improving research literacy of lay people and we hope that this study would be extended and applied to many different settings around the world.

 Given the limitations I would suggest the authors need to be more circumspect In their conclusions about the usefulness of this current checklist given the extremely limited validity checking of it this is a very useful pilot but I would say I was only very preliminary work.

Reply: Thanks a lot for your comments and suggestions. We hope our manuscript now clearly states the limitations of our study and highlights that the assessment is only qualitative and restricted to a small set of perprints.

**Minor**. In figure 2 the "implementation stage" might better be labelled a "piloting stage" - this was really pilot testing the draft checklist out on a number of groups before finalising the checklist, rather than implementation to the wider community.

Reply: We called this stage implementation stage because we wanted to showcase how we implemented the checklist in our teaching and in the interaction with our communications departments without the intention of adapting the checklist afterwards. But of course you are right that piloting is the more common term. However, we already called the sensitivity test a pilot test, and thus decided on “Preliminary implementation stage” as a compromise. We hope that this change is addressing your concern sufficiently.
